# ﻿Single-blind validation of space-based point-source detection and quantification of onshore methane emissions

**DOI:** 10.1038/s41598-023-30761-2

**Published:** 2023-03-07

**Authors:** Evan D. Sherwin, Jeffrey S. Rutherford, Yuanlei Chen, Sam Aminfard, Eric A. Kort, Robert B. Jackson, Adam R. Brandt

**Affiliations:** 1 Department of Energy Science & Engineering, Stanford University, Stanford, CA 94305 USA; 2 ExxonMobil Upstream Research Company, Spring, TX 77389 USA; 3 Climate and Space Sciences and Engineering, University of Michigan, Ann Arbor, MI 48109 USA; 4 Earth System Science, Woods Institute for the Environment and Precourt Institute for Energy, Stanford University, Stanford, CA 94305 USA

**Keywords:** Environmental impact, Natural gas, Energy infrastructure

## Abstract

Satellites are increasingly seen as a tool for identifying large greenhouse gas point sources for mitigation, but independent verification of satellite performance is needed for acceptance and use by policy makers and stakeholders. We conduct to our knowledge the first single-blind controlled methane release testing of satellite-based methane emissions detection and quantification, with five independent teams analyzing data from one to five satellites each for this desert-based test. Teams correctly identified 71% of all emissions, ranging from 0.20 [0.19, 0.21] metric tons per hour (t/h) to 7.2 [6.8, 7.6] t/h. Three-quarters (75%) of quantified estimates fell within ± 50% of the metered value, comparable to airplane-based remote sensing technologies. The relatively wide-area Sentinel-2 and Landsat 8 satellites detected emissions as low as 1.4 [1.3, 1.5, 95% confidence interval] t/h, while GHGSat’s targeted system quantified a 0.20 [0.19, 0.21] t/h emission to within 13%. While the fraction of global methane emissions detectable by satellite remains unknown, we estimate that satellite networks could see 19–89% of total oil and natural gas system emissions detected in a recent survey of a high-emitting region.

## Introduction

Cutting anthropogenic methane emissions is a major near-term greenhouse gas (GHG) reduction option, often with low or negative net cost^1,2^. Recent data from aircraft and satellite surveys suggest that these emissions are substantially larger than previously thought^3–5^. The outsized role of rare, very large point-source emissions has been demonstrated in multiple studies, particularly in fast-growing, oil-rich regions such as the United States Permian Basin^4–8^. Major emissions, with magnitudes of metric tons of methane per hour or higher, have been seen around the world, with large sources identified in oil and gas producing regions for which there were previously little to no published methane data^3^.

Satellite-based methane sensing technologies have the potential to identify large methane emissions from multiple sectors quickly across the globe and flag them for repair or mitigation. Tiered networks of satellite-based methane sensors will allow rapid identification of the largest emissions. The often-intermittent nature of large methane emissions highlights the importance of repeated measurements^5^. Lower-resolution configurations can automatically survey most of Earth’s land area over some regular period of time. High-resolution point-and-shoot satellites, with a smaller targeted field of view, allow more sensitive investigation of specific infrastructure that may be likely to emit methane. Satellites are thus uniquely poised to contribute toward independently assessing compliance with national and international methane reduction commitments such as the Global Methane Pledge^9^.

Growing efforts have therefore arisen to develop satellite-based methane-sensing technologies, moving rapidly from detecting whole-field emissions^10^ to identifying point-source emissions from individual facilities^3,7,11–13^. Large investments are therefore being made in satellite networks due to launch in coming years^14^. In addition, data from some pre-existing satellite networks such as Sentinel-2 and Landsat 8 have been repurposed to detect methane, facilitating retrospective analysis of historical methane emissions across the globe^11,15,16^.

Establishing a multi-stakeholder consensus over the capabilities of methane-sensing satellites is critical for integrating them into national and international methane policy and accounting. For example, efforts by countries to assess and reduce the greenhouse gas intensity of imported fossil fuels could rely on satellites in regions where deployment of ground-based or aerial technologies would be difficult. Other potential uses include domestic identification and assessment of greenhouse gas emissions, and validation of atmospheric models of methane sources and sinks.

Existing satellite validation efforts largely focus on the consistency of quantification estimates across satellites and methods^11,15,17^, or rely on internal and generally unpublished controlled methane release testing. Although independent blind testing has become commonplace for terrestrial and airborne methane sensing technologies, to our knowledge there has until now been no such testing of the methane detection and quantification capabilities of satellites^18–20^. This is largely due to the expense and logistical challenges of performing releases at scales visible from space (100–1000 s of kg of methane per hour).

## Single-blind testing

To provide such validation, we employed a fixed-location single-blind experimental design to test point-source methane sensing systems. Participating teams were aware of the existence, timeframe, and rough location of the test, but were not informed of the size of the emissions released or the precise configuration of equipment on the ground. Large volumes of methane were released from a metered stack, after regassification from a liquified natural gas (LNG) truck. Releases were performed in Ehrenberg, Arizona, United States over a 19-day period from October 16 to November 3, 2021 (see “Materials and methods”).

Metered controlled release volumes—including zero-volume releases—were retained by our team and not given to participating analysts until all estimates were submitted by all teams for all stages of the test. Analysts estimated methane emissions for each overpass, with reporting in compliance with the Advancing Development of Emissions Detection protocol for airplane and satellite systems^21^.

We performed releases during overpasses of five satellites: the commercial GHGSat-C2 (GSC2) and WorldView 3 (WV3) instruments, and the publicly-funded Sentinel-2, Landsat 8 (LS8), and PRISMA satellites. With the exception of GHGSat-C2, none of these satellites was explicitly designed for methane sensing, but their data have instead been repurposed to this end. Analysts first attempted to estimate emissions volumes using available data from satellites and wind reanalysis products. In some cases, multiple teams assessed the same observation from an instrument, giving us the ability to empirically assess variability derived purely from the measured spectra and source quantification algorithms, which participating teams were not required to release. See the SI, Section S2 for the details each team elected to share about their algorithms.

The fact that all tested methane-sensing satellites rely on reflected sunlight introduces design trade-offs between spatial coverage, revisit frequency, and sensor resolution, compounded by the fact that most of these satellites were not designed for methane point source sensing. These tradeoffs are illustrated in Table 1. For example, the TROPOMI instrument of the Sentinel-5P satellite (not included in this test) achieves global coverage with daily revisit time, but primarily detects emissions of 10 metric tons of methane per hour or greater (hereafter t/h, measured on a mass of methane basis unless otherwise stated). This is due to the large effective pixel size of the TROPOMI instrument, roughly 7 × 7 km^3^. Sentinel-2 and Landsat 8 passively cover nearly the entire world’s landmass with per-satellite revisit times of 10 and 16 days, respectively, with teams reanalyzing these data advertising minimum detection limits of 1–3 t/h^11,17^. GHGSat, PRISMA, and WorldView 3 are targeted “point-and-shoot” instruments, facilitating minimum detection levels claimed to be as low as 0.1 t/h at the expense of comprehensive spatial coverage^7,22,23^. In principle, all of these satellites have the theoretical ability to observe over most of Earth’s land area when sufficient solar radiance is present. See supplementary information (SI), Section S1 for further discussion of each satellite.

**Table 1:** Key characteristics of each participating satellite constellation, from lowest to highest swath width, which is roughly proportional to an instrument’s minimum methane detection limit.

Satellite	Coverage	Constellation size	Swath (km)	~ Revisit time (per satellite) (days)	Data availability
GHGSat-C2^22^	Targeted	5 (C1–C5)*	12	14	Commercial
WorldView 3^24^	Targeted	1	13.1	4.5^‡^	Commercial
PRISMA^25^	Targeted	1	3	7	Public
Landsat-8^26^	Global	1	185	16	Public
Sentinel-2^27^	Global	2	290	10	Public

Global coverage refers to a configuration that passively covers most of Earth’s surface over some number of orbits, while targeted coverage refers to a “point-and-shoot” instrument that must be pointed to a particular location.

*GHGSat-C3–C5 were launched after the conclusion of testing.

^‡^For best resolution within 20° off nadir. WorldView 3 has 1-day revisit time at lower guaranteed resolution.

Participating analysis teams include private companies GHGSat^28^ and Kayrros^29^, as well as the government research institution Stichting Ruimte Onderzoek Nederland (SRON)^30,31^ and the Land and Atmosphere Remote Sensing (LARS) group of university researchers from Universitat Politècnica de València (Luís Guanter, Itziar Irakulis Loitxate, Elena Sánchez García, and Javier Gorroño Viñegla^7,15,32,33^) and Harvard University (Daniel Varon^11,13,34^). Each analysis team had the opportunity to submit estimates for all satellites tested, with the exception of GHGSat-C2, to which GHGSat had sole access. See the SI, Section S2 for a description of each team and its members.

Following completion of the testing, methane emission estimates were submitted by participating teams in two rounds in a staged unblinding process. In stage 1 of the assessment, participating teams were provided times of releases and analyzed the associated satellite image data without access to any ground-based meteorological measurements, using only rough coordinates for the release location, accurate to within 150 m. After submitting these stage 1 estimates, teams received unblinded 10-m ultrasonic anemometer wind measurements (1 Hz) from the experimental site and the precise coordinates of the release point. The teams then used the measured wind vector, at 1-s resolution, and release point coordinates to make updated stage 2 estimates to assess resulting accuracy gains. Teams were free to choose how to integrate these data into their estimates and were not required to report the chosen methods. See “Materials and methods” for further discussion of the experimental design and methods employed during testing.

## Results

**Figure 1:**
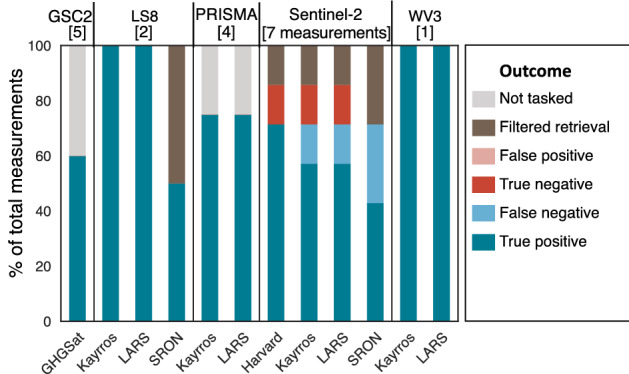
Detection performance by satellite and team. Total number of measurements listed in brackets. For each satellite, most teams correctly detected most emissions as true positives or true negatives (correctly identified non-emissions). In some cases, e.g. two GHGSat-C2 (GSC2) overpasses, the satellite was not tasked and collected no data. In others, e.g. one SRON retrieval of Landsat 8 (LS8), no retrieval was attempted due to image clipping concerns or excessive cloud cover. No teams produced false positives, in which satellites detected methane when none was released.

Of the 49 reported estimates from five teams, 35 (71%) were correctly identified, either as a true positive detection or a true negative in which no methane was emitted or detected (Fig. 1). True positives were 32 (65%) of the total, with 3 (6%) true negatives. Note that true positive rates would fall for a test focused on smaller emissions. Furthermore, there were no false positives, in which a satellite incorrectly reported the presence of methane when no release occurred, so all 4 (8%) mis-identified cases were false negatives. In all cases, emissions seen ranged from metered values of 1.4 [1.3, 1.5, 95% confidence interval] to 7.2 [6.9, 7.6] t/h with the exception of GHGSat, with a minimum metered release level of 0.20 [0.19, 0.21] t/h. A single release value of 0 t/h, given to Sentinel-2, was correctly identified by all three reporting teams as a non-emission event. A total of 6 cases (12%) resulted in filtered retrievals in which no estimate was attempted due either to unfavorable weather conditions, particularly heavy cloud cover, which blocks transmission of the required infrared light, or due to image clipping concerns. See the SI, Figure S17 for sky photographs taken each day, which document cloud cover near the overpass time. Each team used its own filtering criteria, which they were not asked to disclose. This highlights that even in the US desert Southwest, cloud cover can be a limiting factor for satellites. In 4 cases (8%), satellites passed over but were not tasked due to internal scheduling issues on the satellite side, with no data collected as a result. Note that these detection results are identical for stages 1 and 2 of the analysis, which differ only in quantification estimates.

**Figure 2:**
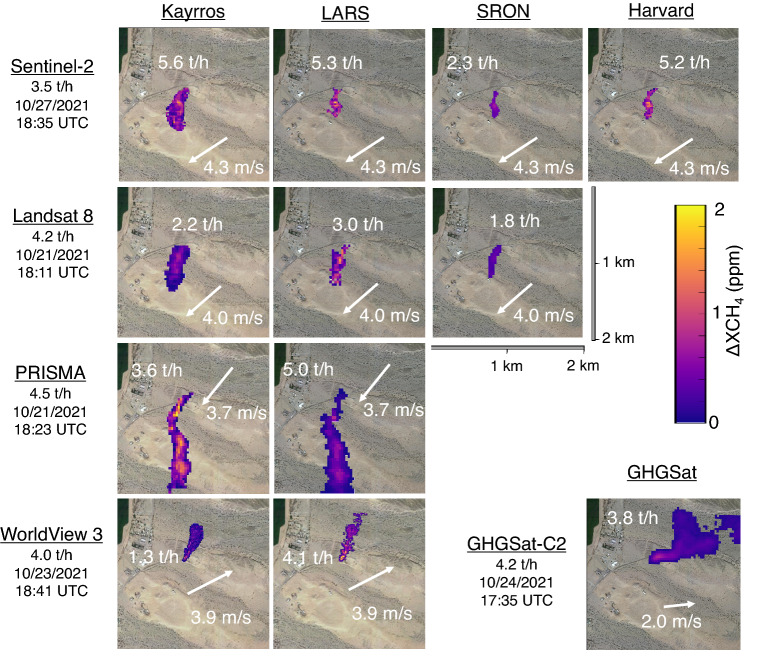
Visualization of detected emissions for all satellite-team combinations for mean metered release values in the 4 t/h approximate range (3.6–4.5 t/h). The true measured emissions rate and timestamp are given below satellite name. Mean estimated volume from each team/satellite pair, as well as measured 1-min average 10 m wind speed and direction, are superimposed on the corresponding picture. Note that measured time trends in wind speed and direction can cause irregular plume shapes, e.g. for PRISMA and GHGSat-C2, which are also cut off in these images due to plume length. See the SI, Section S7 for full plume images. Surface imagery © 2021 Google Earth.

While methane-release volumes were randomized, each satellite was given at least one release in the 4 t/h range (3.6–4.5 t/h), measured as the average flow rate over 300 s prior to overpass, accounting for uncertainty as described in “Materials and methods”. All teams were able to correctly detect these emissions, with false-color methane plume images shown in Fig. 2, with each team applying the same color scale for methane enhancement. Although each image shows an emission of similar magnitude, the plume appears much larger and longer for more sensitive instruments, such as GHGSat-C2 and PRISMA than for less sensitive instruments, such as Sentinel-2 and Landsat 8. Estimated mean emission rate for each team and 10-m one-minute average measured wind speed and direction are inset in white. Average and standard deviation for wind speed and direction values on the selected days are as follows: Sentinel-2, 4.3 [σ = 0.7] m/s at 57° [σ = 15°]; Landsat 8, 4.0 [σ = 0.9] m/s at 49° [σ = 8°]; PRISMA, 3.7 [0.7] m/s at 39° [σ = 5°]; WorldView 3, 3.9 [σ = 0.7] m/s at 243° [σ = 10°]; GHGSat, 2.0 [σ = 0.4] m/s at 264 [σ = 11°]. Average wind speed and direction values, including uncertainty, are available for all satellite measurements in the supplied data and code repository.

### Quantification performance is similar to aircraft

**Figure 3:**
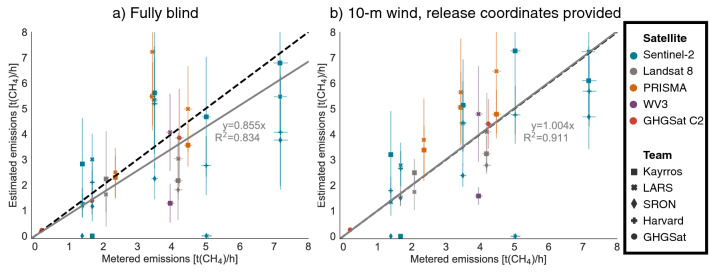
Quantification performance of methane emissions estimates by satellite and team. Metered emissions compared with single-blind estimates for each overpass with successfully reported data, with 1-sigma X and Y error bars. (**a**) Fully blind stage 1 results using modeled wind speed estimates. (**b**) Stage 2 results using 10-m wind speed and direction measurements and the point source location of the plume. Percent error in both cases is similar to aircraft-based methane remote sensing methods. The grey solid line is a linear fit with the intercept fixed at zero, with slope and uncentered R^2^ displayed. The black dashed lines denote exact 1:1 agreement.

The selected emission rates ranged from 0.20 [0.19, 0.21] t/h, analogous to a large process emission from a liquids unloading event at an oil and gas production site^35^, to 7.2 [6.9, 7.6] t/h, analogous to a large unlit flare^4^. For all detected emissions, mean estimates for all satellite-team combinations are between – 68 and 110% of the metered value (Fig. 3; see also SI, Section S7), with 75% of nonzero estimates falling within ± 50% of the metered value. This performance is similar to error demonstrated by aerial remote sensing technologies, such as Kairos Aerospace, with 86% of nonzero single-blind estimates falling within ± 50% of the metered value (described in detail in the SI, Section S3)^18^. See the SI, Section S7 for error summary statistics by satellite and team. Note the presence of non-trivial error in metered values, due to metering uncertainty, flow variability, and uncertainty in gas composition, described further in^36^.

In stage 2 of the test, teams produced updated results using measured 10-m wind data from an on-site ultrasonic anemometer as well as precise coordinates of the single, stationary release location, though still blind to released volumes. These estimates are 20% higher on average than stage 1 estimates, roughly matching the average 19% underestimate in the modeled winds used in stage 1 (in emissions remote sensing methods, higher winds with the same observed methane enhancement imply greater methane flux). Applying an ordinary least squares linear fit to all quantified emissions, with the intercept set to zero, we see substantial improvement in slope, rising from 0.855 [0.715, 0.995] in stage 1 to 1.004 [0.889, 1.120] in stage 2, almost an exact one-to-one average agreement, though still with substantial noise (Fig. 3). After incorporating on-site wind measurements, the uncentered R^2^ increases from 0.834 to 0.911, implying a tighter fit. Note that this fit treats each estimate from each team as an independent datapoint and that uncentered R^2^ values from a linear fit with a zero intercept are not directly comparable to R^2^ values from regressions with a nonzero intercept. See the SI, Section S5 for further discussion of the reasons for this regression specification, which is more physically interpretable than a standard floating-intercept regression but may introduce modest upward bias in the slope of order 5%. This improved average linear fit does not translate to lower error for each individual satellite, as shown in Table S5. Note that these results suggest that error in average wind speed largely explains overall error in stage 1 quantification estimates, implying that quantification accuracy depends more on wind speed accuracy than knowledge of the precise emission source location. See the SI, Section S7 for additional regression results.

Although errors for larger emissions decrease in stage 2, errors increase for some smaller emissions, resulting in a percent error distribution that is similar across stages, as shown in the SI, Section S7 alongside full regression results. These results highlight the likely importance of wind speed and emission source location as sources of uncertainty in current satellite-based methane quantification methods, alongside other algorithmic uncertainties.

Note that in general practice, emission location information is not known at all, or only the location of candidate sources is known. Knowledge of potential source locations may facilitate more effective discrimination between true emissions and false positive signal artifacts, improving detection performance, particularly for smaller emissions, as illustrated in Figures S6–S16 in the SI, Section S7, which show masked methane plumes, as in Fig. 2, alongside raw, unmasked methane enhancement estimates for the full scene, which often include artifacts due to the reflectivity profile of some ground features. Further testing is needed, without notifying teams of the emission location, to more comprehensively assess the effect of emission location information on detection and quantification performance.

Confidence intervals provided by teams appear to be slightly overconfident, with the metered value lying within 1-sigma error range 56% of the time, instead of the 67% one would expect for perfectly calibrated confidence intervals. Provided 95% confidence intervals, shown in the SI, Section S7, contain the true value 88% of the time. In addition, percent quantification error appears to rise for lower emission rates, particularly for Sentinel-2, as is visible in Fig. 2 and shown further in Figures S3 and S4. The largest error for the smallest Sentinel-2 retrieval is + 103%, among the largest in the dataset. Note that these statistics combine results from multiple teams and satellites, and as such represent an overall sense of the performance of satellite systems as a class of methane sensing technology. With a larger dataset, it will be possible to determine calibration for individual satellites and analysis teams, but this will require significant additional experimental work because of the large number of datapoints that would be needed.

These results provide an approximate first upper bound for the minimum detection capabilities of each instrument. GHGSat detected the smallest emission of the campaign, at 0.197 [0.187, 0.208] t/h and maintained stage 1 error between − 17% and + 13% for each of the three measurements with valid data collection. See the SI, Section S7 for all quantification error summary statistics by satellite and team. Three of the four teams analyzing Sentinel-2 data detected the smallest emission, metered at 1.4 [1.3, 1.5] t/h, which is close to the expected minimum detection threshold of the method^11^.

## Discussion

How useful might these satellites be in detecting emissions for control and mitigation? Lauvaux et al. recently estimated that oil and natural gas emissions visible to the TROPOMI instrument, (detection limit of > 10 t/h), represent 8–12% of estimated global emissions from the sector^3^.

At present, the full distribution of methane emissions from oil and natural gas systems (or other methane-emitting industries) is not sufficiently characterized in most regions to determine the fraction of total regional emissions visible to satellites. We can, however, use data from the Chen, Sherwin et al. 2018–2020 comprehensive aerial survey of the New Mexico Permian Basin to simulate the capabilities of satellites in one high-emission basin, emitting an estimated 9.4% [5.9%, 12.7%] of total natural gas production in this region^4^. A comprehensive survey of the same area of New Mexico over the same time period with wider-area satellites similar to Sentinel-2 or Landsat 8, with a minimum detection threshold of 1–5 t/h, would see 19–47% of gross detected emissions volume (counting each detected plume as a separate measurement) estimated by Chen, Sherwin et al. These emissions come from 11 to 117 out of a total of 1985 individual detected emissions from over 26,000 active oil and gas wells and associated midstream infrastructure^4^. A comprehensive survey with a sufficiently large network of point-and-shoot satellites with a minimum detection threshold of 0.1–0.5 t/h (such as GHGSat, PRISMA, or WorldView-3) would see an average of 61–89% of gross detected emissions volume, from 258 to 1182 individual detected emissions^4^. Note that satellite surveys of lower-emitting regions would likely find a smaller fraction of total emissions. See the SI, Section S6 for the details of this regional survey simulation.

Importantly, improved technology is on the way. At least eight constellations with point source imaging capability are in orbit or scheduled for launch by the end of 2023^16^. The constellation of satellites from Carbon Mapper has a designed detection limit of 0.05–0.15 t/h, with revisit times of 1–7 days at full constellation deployment^37^. If quantification accuracy is similar to what we observe in the above real-world blind tests (especially at observed GHGsat accuracy), then we will be able to find and quantify a substantial fraction of global methane emissions volume from oil and gas and other point sources, anywhere on the planet, on daily-to-weekly scales. The complementary MethaneSAT project from the Environmental Defense Fund will provide independent coverage using another advanced purpose-designed methane sensor, with both point source and wide-area methane flux estimation capabilities at somewhat larger spatial scales^38^. A total of eight area flux mapping satellites are scheduled for orbit by 2027^16^.

### Toward a globally accepted methane satellite validation regime 

It is increasingly clear that satellite-based sensing efforts can play an important role in understanding methane emissions across borders. The adoption and acceptance of this approach will depend in part on the confidence all parties involved place in the technology itself. The five satellites we tested correctly detected most emissions and had no false positives, bolstering confidence in these techniques, at least in cases with a pre-identified potential emission source. Despite the need for larger plumes in order to obtain a signal (due to a comparatively large minimum detection threshold), the quantification accuracy for emissions that were detected is similar to airplane-based technologies. Importantly, the only purpose-designed methane satellite we tested, GHGsat, achieved quantification accuracy better than ± 20% in each of its studied emissions.

This study outlines a first step toward ongoing, operational blind testing of satellites quantifying methane point sources. Significantly more blind testing—like we performed above—is needed to ensure rapid uptake and trust. Such tests should include offshore releases, undisclosed location testing, onshore releases with varying weather and ground cover, diffuse-source releases, and possibly multi-source releases. As the technology evolves, the validation task remains similar scientifically, but becomes increasingly important. Our test was conducted under near-ideal land surface and wind speed conditions, and teams were aware in advance of the approximate emission location. Rigorous characterization of the detection and quantification capabilities of airplane-based methane remote sensing systems requires of order 100 measurements, as in^18^, more than 10 times the number for any satellite in this study. Achieving similar evaluation of existing and emerging satellites will thus require further data collection. Satellites are already providing invaluable insights into methane emissions from multiple industries^7,12^. Additional independent empirical validation under a range of field conditions will enable satellites to play an even greater role in reducing global methane emissions from oil and natural gas and industries including waste management, agriculture, and mining.

## Materials and methods

### Materials

For the duration of testing, our controlled methane release apparatus was located near Ehrenberg, Arizona, on the border of Arizona and California in the United States, at approximately [33.630637°, − 114.487755°]. These are the coordinates initially given to all satellite operators.

**Figure 4:**
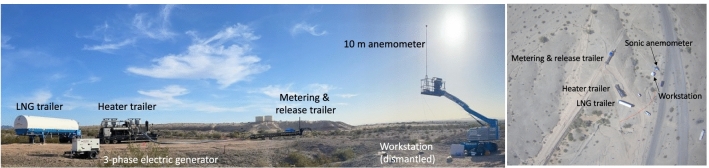
The experimental setup in panorama (left). Liquefied natural gas was reheated before flowing through a metering and release trailer. An aerial photograph of the site (right) [courtesy of Bridger Photonics Inc.]. Note that the release trailer is ~ 45 m from liquefied natural gas (LNG) trailer and ~ 45 m from workstation and anemometer. Adapted with permission from^36^.

**Figure 5:**
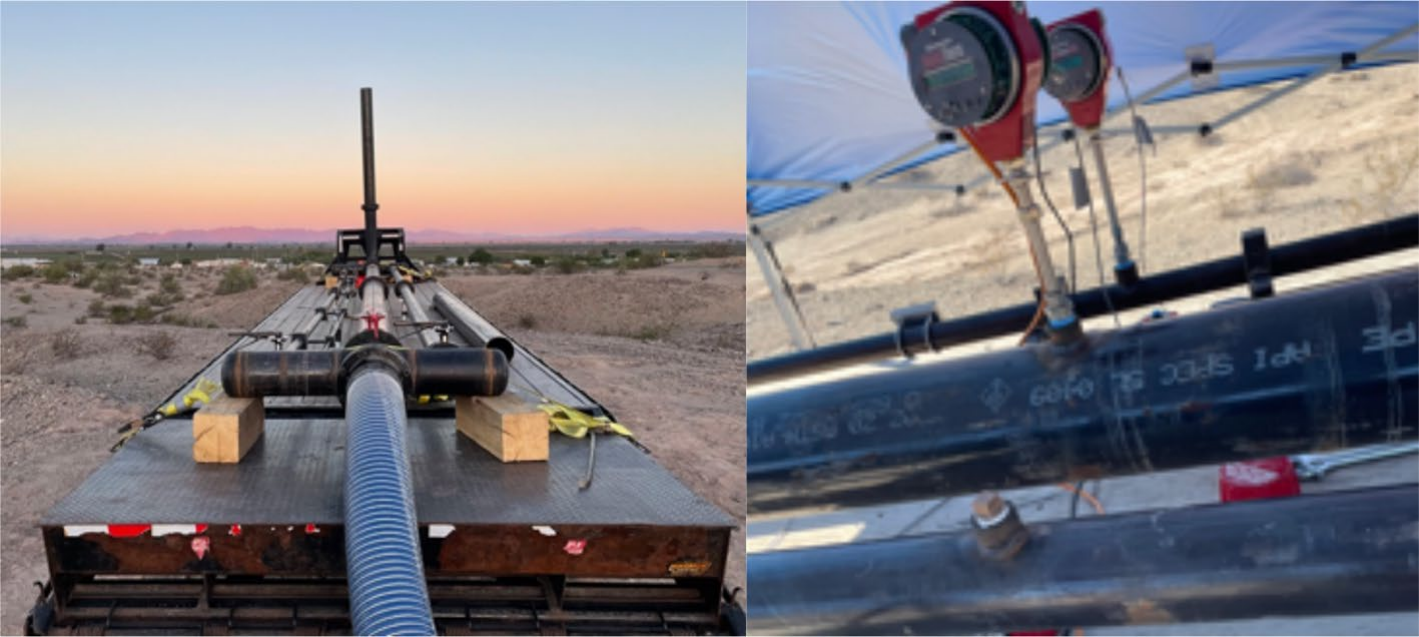
Metering and release trailer (left) has four pipes (0.5-in., 2-in., 4-in., and 8-in.), each leading to a distinct metering location allowing a different dynamic range of release volumes. The three larger pipes used a QuadraTherm 640i insertion meter (right), with upstream 8.5 m straight run of pipe to ensure sufficient flow stabilization for all pipes. The 0.5-in. pipe was not used in this study.

The methane source was a trailer of liquefied natural gas, shown in Fig. 4, which was reheated from roughly – 160 °C to temperatures generally ranging from 30 to 65 °C, within the rated tolerance of our Sierra QuadraTherm 640i meters^39^. For the October 19 Sentinel-2 overpass, a heater issue resulted in a gas temperature of − 38 °C, still within range but close to the minimum rated temperature of − 40 °C. The gas was then transmitted to the metering and release trailer via an 8″ shipping hose at an exit pressure of roughly 80–150 psi (0.55–1.03 Mpa), passing through one of four possible metering locations before release through a single stack located at [33.630645°, − 114.489150°], at a release height of 5 m above ground level, shown in Fig. 5. This testing setup roughly mimics an unlit flare on an oil and gas production site or other facility.

This experiment used only the 2-in., 4-in. and 8-in. pipes to achieve the desired release volumes. Using QuadraTherm 640i insertion meters, the maximum achievable release rate for the 8-in. pipe is 416,400 standard cubic feet per hour (scfh), or 8.0 t/h for pure methane. For the 4-in. pipe, the maximum release rate is 106,080 scfh or 2.0 t/h for pure methane, falling to 27,960 scfh or 0.5 t/h for the 2-in. pipe^40^. These values fall by roughly 20% for the meter used for the November 3 Sentinel-2 release. See^36^ for further detail.

### Safety

All natural gas equipment fabrication, transportation, assembly, and operation was conducted by personnel from Rawhide Leasing, a gas services contractor. No Stanford personnel operated natural gas release equipment. The research workstation, at which Stanford researchers coordinated field operations and data collection procedures, was ~ 45 m away from all equipment through which natural gas flowed and researchers maintained a distance of at least 30 m from this equipment during active releases (e.g. when walking).

In addition, Stanford researchers continuously monitored plume dissipation in real time using a FLIR GasFinder 320 infrared camera and paid continuous attention to olfactory signals from the odorized gas. The infrared camera showed clear plume dissipation well before reaching any on-site personnel, particularly because the emission source was elevated and gas often exited at a high vertical velocity, accelerating natural methane lofting. Stanford researchers rarely detected gas by smell during the test period.

### Data logging 

Stanford researchers logged data from the gas flow meters using a Eurotherm Nanodac automatic data logger, with an iPhone-based Zoom livestream of the digital meter display as a backup. All data in this study use the automatic data logger except the GHGSat and PRISMA measurements on October 16 and the Landsat 8 measurements on October 21, which use Google optical character recognition to extract a flow rate time series from the Zoom livestream^41^. Data from the data logger and the Zoom livestream of the digital meter display agree to within 1%.

### Data collection procedures

All satellite-coincident releases began at least 15 min before the scheduled satellite overpass time, provided by participating teams. Initial releases began as much as 30 min in advance. After receiving initial plume images from satellite participants, it became clear that a methane plume moving at the average wind speed would stabilize well within 15 min at the release volumes in this study. In practice, all emission rates stabilized within 11 min of the satellite overpass.

A Rawhide Leasing technician set all release levels based on hand signals from the Stanford team, which requested adjustments to release volumes based on the Zoom livestream of the digital meter readout. Due to the engineering properties of the gas release system, flow rates often varied somewhat during these releases. Stanford personnel aimed to maintain rates within ± 10% of the targeted value and requested adjustments as needed from Rawhide Leasing personnel.

Over the course of the experiment, we tested the GHGSat AV and Bridger Photonics aerial methane sensing systems^36,42^, both of which are more sensitive than any of the satellites tested. These aircraft, which also surveyed the surrounding area in the course of data collection, did not detect any methane sources outside our test site. This strongly suggests that our results are free of interference from any significant confounding methane sources.

### Flow rate uncertainty

Sources of uncertainty in measured flow rates include variability in actual flow rates (captured as the standard deviation of metered flow over a 5-min period), rated meter uncertainty, error introduced by variation in meter placement during installation, and uncertainty in gas composition, which can vary even for a consistent supplier. Natural gas composition for these releases ranged between 95.22 and 96.27% methane, in the range for which the QuadraTherm meters were calibrated^36^. We propagate these sources of error into our metered values using a Monte Carlo-based approach with 10,000 iterations using code listed in our Data availability statement. See^36^ for further discussion of sources of metering uncertainty and the Monte Carlo approach, as well as detailed LNG gas composition data.

The 5-min averaging period used to compute flow variability is based on the fact that a plume traveling with a relatively slow average wind speed of 2 m/s would traverse 600 m within 5 min (300 s). By this distance, much of the originally emitted methane has likely dissipated into background concentrations, with the bulk of the methane enhancement detected by a satellite remaining closer to the release point. A sensitivity analysis in the SI, Section S7 shows that switching this averaging period to 60 s or 600 s has a minimal effect on the results.

### Experimental design

This single-blind field trial employed a two-stage experimental design.

Stanford personnel initially released metered quantities of methane from the Arizona test site via procedures described above and in^36^. The Stanford ground team and contract personnel operating equipment communicated no information to participating teams regarding metered flow rates, metered wind speed or direction, or the precise location of ground-based equipment. In addition, participating teams were not informed of the details of the equipment or its configuration, e.g. the use of liquefied natural gas v. compressed natural gas (which Stanford used in previous tests), or the diameter of the pipes and hoses involved.

Thus, all teams produced stage 1 estimates using only information from their existing systems, including satellites and available wind reanalysis products, based on coordinates that identified the release location to within 0.15 km, at the coordinates [33.630637°, − 114.487755°].

After each team submitted finalized stage 1 estimates, we provided 10 m wind speed and direction readings from our on-site ultrasonic anemometer (shown in Fig. 4), as well as the precise coordinates of the release stack, [33.630645°, − 114.489150°]. All teams submitted both stage 1 and stage 2 estimates on a timeline described below in Table 2. Note that turnaround time in these tests may not be representative of field performance.

**Table 2:** Data submission timeline by stage for each team.

Operator	Submitted stage 1	Received wind and location data	Submitted Stage 2
GHGSat	1-Dec-21	8-Dec-21	28-Feb-22
Kayrros	26-Nov-21	13-Jan-22	28-Feb-22
LARS	11-Nov-21	11-Feb-22	23-Feb-22
SRON	25-Feb-22	25-Feb-22	28-Feb-22
Harvard	2-Feb-22	23-Feb-22	25-Feb-22

## Supplementary Information

Supplementary Information.

## Data Availability

All data and code required to reproduce the figures and analysis in this paper are available on GitHub at https://github.com/esherwin/SatelliteTesting. The full time-resolved profile for all metered methane emissions in this paper, as well as the code for computing time-integrated emission estimates with uncertainty, are available at https://github.com/JSRuthe/Controlled_Release_2021.

## Abbreviations

EPA: Environmental Protection Agency
GHG: Greenhouse gas
GSC2: GHGSat-C2 (satellite)
LS8: Landsat 8
LARS: Land and Atmosphere Remote Sensing
PRISMA: PRecursore IperSpettrale della Missione Applicativa
SI: Supplementary information
SRON: Stichting Ruimte Onderzoek Nederland
TROPOMI: TROPOspheric Monitoring Instrument
WV3: WorldView-3
