# Supplementary material for: ﻿Single-blind validation of space-based point-source detection and quantification of onshore methane emissions
Source: Sci Rep. 2023 Mar 7;13:3836. doi: 10.1038/s41598-023-30761-2 (PMC9992358; doi:10.1038/s41598-023-30761-2)
Supplement: Supplementary file 1 — Supplementary Information. [file 41598_2023_30761_MOESM1_ESM.pdf]

# Supplementary information for “Single-blind validation of space-based point-source detection and quantification of onshore methane emissions”

**Authors:** Evan D. Sherwin[1,*], Jeffrey S. Rutherford[1], Yuanlei Chen[1], Sam Aminfard[2], Eric A. Kort[3], Robert B. Jackson[4], Adam R. Brandt[1]

**Author Affiliations:**

[1] Department of Energy Resources Engineering, Stanford University, Stanford, California 94305, United States

[2] ExxonMobil Upstream Research Company, Spring, Texas 77389, United States

[3] Climate and Space Sciences and Engineering, University of Michigan, Ann Arbor, Michigan 48109, United States

[4] Earth System Science, Woods Institute for the Environment and Precourt Institute for Energy, Stanford University, Stanford, California 94305, United States

[*] Correspondence: evands@stanford.edu

**Table of contents**

S1. Participating satellites
S2. Participating teams
S3. Estimating the fraction of Kairos controlled releases with error <±50%
S4. Notes on linear fitting for quantification evaluation
S5. Determining what satellites would see in the New Mexico Permian Basin
S6. Supplementary results

# S1 Participating satellites

Six satellites commonly used for methane sensing were available to collect measurements during the study period of October 16-November 3, 2021. This included targeted satellites GHGSat, WorldView-3, and PRISMA, which must be tasked to focus on a particular area, as well as global-coverage satellites Landsat-8, Sentinel-2, and Sentinel-5P (TROPOMI), which passively collect data from nearly all inhabited areas of the world [22,24–27,44].

Table 1 summarizes the spectral resolution, spatial coverage, constellation size, swath width, revisit time, and data availability (commercial or public). We opted not to test Sentinel-5P due to its lower resolution, which corresponds to a minimum methane detection limit likely above the capabilities of our equipment. Below, we describe each satellite in more detail.

Note that only the GHGSat instruments were originally designed for the primary purpose of detecting and quantifying methane emissions. With the remaining satellites, researchers have developed methane retrieval techniques based on existing data [3,7,11,32].

## S1.1 Sentinel-2

The two-satellite Sentinel-2 constellation consists of Sentinel 2A, launched June 23, 2015, and Sentinel 2B, launched March 7, 2017 as part of the European Union's Copernicus program [45]. The satellites operate in the same 10-day polar orbit offset by 180°, resulting in 5-day revisit times at the equator, falling to 2-3 days at mid-latitudes. Each satellite collects data for all inhabited areas of the world each orbit with a 290 km swath with thirteen spectral bands in the short-wave infrared (SWIR) and Visible to Near Infrared ranges. This includes four bands at 10 m resolution, six bands at 20m resolution (including Band 12 at 2190 nm in the SWIR range), and three bands at 60m resolution [46]. All data from Sentinel-2 are publicly available at [47].

## S1.2 Landsat 8

Launched on February 11, 2013, the Landsat 8 satellite is the product of a collaboration between the National Aeronautics and Space Administration (NASA) and the United States Geological Survey (USGS), both agencies of the United States government. This instrument has global coverage, collecting data for all inhabited areas of the world every 16 days with a 185 km swath. The satellite hosts a 9-band operational land imager, including two SWIR bands at 1570-1650 nm and 2110-2290 nm, as well as four visible bands, all at 30 m resolution. An onboard thermal infrared sensor also collects two bands at 10,600-11,190 nm and 11,500-12,510 nm, both at 100 m resolution. All data from Landsat 8 are publicly available at [48].

## S1.3 PRISMA

Launched March 19, 2019, the PRISMA (PRecursore IperSpettrale della Missione Applicativa) satellite is a product of the Italian Space Agency (ASI), contracting through Orbitale Hochtechnologie Bremen (OHB) Italia S.p.A. This targeted hyperspectral instrument uses spectral bands ranging from 400-2,500 nm with a 30 km swath, operating with a 7-day maximum revisit frequency. Data from PRISMA are publicly available, and the satellite can be tasked upon request [25].

### S1.4 **WorldView-3**

Launched August 13, 2014, the WorldView-3 satellite is owned and operated by United States-based company Maxar. This multispectral instrument measures in one panchromatic band, eight multispectral bands in the visible near infrared range, eight SWIR bands (1195-2365 nm), and twelve bands covering clouds, aerosols, vapors, ice, and snow. This targeted instrument has a 13.1 km swath and a revisit frequency of 4.5 days at 20° off-nadir for maximum resolution [24].

WorldView-3 operates commercially, its data archives as well as tasking are available to scientific researchers for select proposals [49].

### S1.5 **GHGSat-C2**

The GHGSat-C2 satellite is one of two instruments launched by the Canada-based private company GHGSat at the time of this test. GHGSat-C2 was launched on January 24, 2021, following the launch of its counterpart, GHGSat-C1, On September 2, 2020. The precursor GHGSat-D satellite was launched on June 22, 2016. Several additional satellites are scheduled to launch in the coming years, with a goal of achieving a 10-satellite constellation by 2023 [22].

GHGSat-C1 and -C2 each complete 15 orbits per day, with a 14-day repeat cycle. Each satellite is equipped with a multispectral Wide-Angle Fabry-Perot (WAF-P) Imaging Spectrometer, focusing on a proprietary combination of unpolarized short-wave infrared frequencies from 1630-1675 nm at 25m spatial resolution, as well as a secondary VIS-1 Visible Sensor in the optical frequency range at <20m spatial resolution. The sensor has a 12x12 km field-of-view, which can be targeted toward a desired location. GHGSat claims a detection threshold of 0.1 t($CH_4$)/h at 3 m/s winds, with methane column density precision at 1% of background [22].

GHGSat operates commercially, but offers access to data archives as well as tasking to scientific researchers for select proposals [50].

### S1.6 **Sentinel-5P (TROPOMI)**

The Sentinel-5 Precursor (Sentinel-5P) satellite was launched October 13, 2017, also as part of the European Union's Copernicus program [51]. This satellite offers full daily coverage for latitudes greater than 7° or less than -7° and over 95% coverage for remaining latitudes, with a 2600 km swath and 7 km pixels [52]. The onboard TROPOspheric Monitoring Instrument (TROPOMI) includes four spectrometers, each with two spectral bands, including two in the ultraviolet, two in the visible range, two in the near infrared, and two SWIR bands [53]. All data from Sentinel-5P are publicly available at [47]. Due to the low spatial resolution of the instrument, its estimated methane detection threshold is approximately 10 t($CH_4$)/h, too large to test with our equipment in this study [3].

## S2 Participating teams

Five teams participated in this single-blind study, each using data from a subset of the five participating satellites.

We invited all teams of which we were aware that estimate methane emissions from any of the five participating satellites.

Each team was given the option to produce methane retrievals for up to five participating satellites. GHGSat was the only company with access to data from GHGSat-C2 and was thus the only team able to produce an estimate from that satellite, as shown in Table S1.

Table S1. Satellites (columns) analyzed by each team (rows). The final column is the reported source for 10 m wind data for fully blind estimates.

| Team | GHGSat-C2 | Landsat 8 | PRISMA | Sentinel-2 | WorldView-3 | Wind source |
|---|---|---|---|---|---|---|
| GHGSat | X | | | | | GEOS-FP |
| Kayrros | | X | X | X | X | ECMWF ERA5 |
| SRON | | X | | X | | ECMWF ERA5, GEOS-FP |
| LARS | | X | X | X | X | GEOS-FP |
| Harvard | | | | X | | GEOS-FP |

In fully blind stage 1 estimates, all teams used wind reanalysis data from either NASA Goddard Earth Observing System-Fast Processing (NASA GEOS-FP) at 10 m, Fifth generation European Centre for Medium-Range Weather Forecasts Atmospheric Reanalysis of the global climate (ECMWF ERA5), or both [54,55].

## S2.1 Kayrros

Kayrros is a private company specializing in reanalysis of public and private satellite data, with a major area of focus in remote sensing of methane. Kayrros produced estimates for all satellites except GHGSat-C2.

Kayrros retrievals for all satellites relied on methods derived from the algorithm introduced in Varon et al. 2018 [35], the molecular spectroscopic database introduced in the HITRAN2020 model [56], and the LOWTRAN 7 atmospheric transmittance and background radiance model [57]. See the “Performer Info” tab of the Kayrros reported data spreadsheets (available in the GitHub repository) for further detail.

## S2.2 Land and Atmosphere Remote Sensing group (Universitat Politècnica de València)

Researchers Prof. Luís Guanter, Prof. Elena Sánchez García, Itziar Irakulis Loitxate, and Javier Gorroño Viñegla of Universitat Politècnica de València in the Land and Atmosphere Remote Sensing (LARS) group in Spain produced estimates for all satellites except GHGSat-C2.

LARS researchers did not report the details of their retrieval algorithms in this study but did so in other studies. Irakulis-Loitxate et al. used a matched filter-based method for PRISMA retrievals in [33], and for Sentinel-2 and Landsat 8 retrievals in [32]. Sánchez-García et al. [23] apply a retrieval method derived from Frankenberg et al. 2016 and Varon et al. 2018 [35,58] to estimate methane emission rates using WorldView-3.

### S2.3 **Stichting Ruimte Onderzoek Nederland (SRON)**

SRON is the Dutch government space agency, which has a significant focus on remote sensing of methane emissions. Dr. Sudhanshu Pandey produced estimates for Sentinel-2 and LandSat 8 on behalf of SRON.

SRON retrievals relied on the multi-band–multi-pass integrated mass enhancement methane retrieval method introduced in Varon et al. 2021 [11]. See the "Performer Info" tab of the SRON reported data spreadsheets for further detail.

### S2.4 **Harvard University**

Dr. Daniel Varon of Harvard University developed the first method for estimating methane emissions from Sentinel-2 data [11]. Dr. Varon produced estimates for Sentinel-2 only.

Dr. Varon used a modified version of the algorithm described in Varon et al. 2021 [11], adding concepts from Ehret et al. 2022 [17]. See the "Performer Info" tab of the Harvard reported data spreadsheet for further detail.

### S2.5 **GHGSat**

GHGSat is a private company specializing in remote sensing of greenhouse gas emissions. GHGSat owns a constellation of satellites, currently including GHGSat D as well as the more recent GHGSat-C1 and-C2 instruments, with further satellites scheduled for launch in coming years [14]. GHGSat also produces estimates of methane emissions from other satellites, including Sentinel-2, but opted not to do so for this study, in part due to time constraints as the Stanford team did not realize this possibility until one month before the unblinding deadline.

Firmware installed on the GHGSat-C2 instrument was version 10.9.3-gb41c76f, using observation script N08AEB15.GSB. Methane retrievals were then conducted using toolchain version 8.23, via the ghg-ops-srr v0.9.1 source rate retrieval algorithm. See the "Performer Info" tab of the GHGSat reported data spreadsheet for further detail.

## S3 Estimating the fraction of Kairos controlled releases with error <±50%

We use the results of controlled release testing from [18] to compute the fraction of Kairos estimates that fell within ±50 of metered values. As in the paper, we assume natural gas is 93.5% methane (based on local gas composition reports from the utility from which we purchased the natural gas) and use wind speeds measured from the cup wind meter, which was present for all measurements [18]. Replication code is available in the GitHub repository.

## S4 Notes on linear fitting for quantification evaluation

A common metric in controlled release evaluation of sensing technologies is a linear regression fit, with the metered emission rate on the x-axis and estimated values on the y-axis [18,19,43]. If the slope of this line is close to exact 1:1 agreement, this suggests a technology produces estimates that are unbiased on average. A high $R^2$ value indicates the extent to which a linear fit accurately describes variation in the data.

In practice, the x and y error profiles observed in methane controlled release testing tend to deviate from the underlying assumptions of the standard Ordinary Least Squares (OLS) regression that has been standard in the methane sensing evaluation literature. In particular, OLS assumes that:

1. Errors in the y-direction are homoscedastic, with their average magnitude not dependent on the corresponding value of the x variable.
2. x-values are known without significant error.

In addition, unlike many other analyses, we force the y-intercept to zero for reasons related to our relatively small sample size described in this section.

We address these concerns below.

## Impact of normally distributed percent errors on fitted slope

For methane remote sensing technologies, the average estimated value ideally has a roughly linear relationship with the metered emission rate, with an approximately normal distribution of errors in percentage terms [18,43].

In this case, the data-generating process thus has the functional form:

$$y_i = \beta x_i N_i(1, \sigma) \; + \; \beta_o \tag{1}$$

Where $x_i$ represents the true emitted value of release $i$, $y_i$ represents the sensing technology's measurement of release $i$, $\beta$ is a linear scaling factor to capture any average bias in the measurement technology, and $\beta_o$ is a constant y-intercept value. Here, $N_{y,i}(1, \sigma)$ is a random draw from a normal distribution with mean 1 and standard deviation $\sigma$. This introduces normally distributed percent error in the y-direction with standard deviation $\sigma$, proportional to the value of $x_i$ into $y_i$.

Combining all $y_i$ values into vector form, we see that:

$$Y = \boldsymbol{\beta}^T \theta_y^T X \tag{2}$$

Where $X$ is the matrix of $x$ values, with each $x_i$ appearing in column 1 row $i$, with a second column of ones in this case. $Y$ is a vector of $y$ values, with each $y_i$ appearing in row $i$. $\theta_y$ is a matrix with two columns, the first of which is the $N_{y,i}(1, \sigma)$ values and the second of which is a vector of ones. $\boldsymbol{\beta}^T = [\beta, \beta_o]$, a vector representation of these coefficients.

Recall that for a collected dataset the ordinary least squares estimator for the vector of $\beta$ and $\beta_o$ coefficients, $\widehat{\boldsymbol{\beta}}$**,** is computed using the following formula [59]:

$$\widehat{\boldsymbol{\beta}} = (X^T X)^{-1} X^T Y \tag{3}$$

Inserting equation (2), we see that:

$$\widehat{\boldsymbol{\beta}} = (X^T X)^{-1} X^T \boldsymbol{\beta}^T \theta_y^T X \quad (4)$$

Note that the only term in this equation that is dependent on $y$ is $\theta_y^T$. We can thus compute the average with respect to $y$ as follows:

$$E_y[\widehat{\boldsymbol{\beta}}] = E_y[(X^T X)^{-1} X^T \boldsymbol{\beta}^T \theta_y^T X] \quad (5)$$

$$= (X^T X)^{-1} X^T \boldsymbol{\beta}^T E_y[\theta_y^T] X \quad (6)$$

Recall that the $\theta_y^T$ is a matrix whose values are all of the form $N_{y,i}(1, \sigma)$ or 1. Thus, $E_y[\theta_y] = \mathbf{1}$. Thus,

$$E_y[\widehat{\boldsymbol{\beta}}] = (X^T X)^{-1} X^T \boldsymbol{\beta}^T E_y[\theta_y^T] X \quad (7)$$

$$= (X^T X)^{-1} X^T \boldsymbol{\beta}^T \mathbf{1}^T X \quad (8)$$

$$= (X^T X)^{-1} X^T \boldsymbol{\beta}^T X \quad (9)$$

It is trivial to demonstrate that $(X^T X)^{-1} X^T \boldsymbol{\beta}^T X = (X^T X)^{-1} X^T X \boldsymbol{\beta} = \boldsymbol{\beta}$

Thus, under the above assumptions,

$$E_y[\widehat{\boldsymbol{\beta}}] = \boldsymbol{\beta} \quad (10)$$

This proves that unbiased, normally distributed noise in $y$ whose magnitude scales as a percentage of the corresponding $x$ value does not, on average, introduce bias into the fitted slope using OLS.

This $y$-direction heteroskedasticy may, however impact the estimates of the uncertainty surrounding the fitted $\boldsymbol{\beta}$ coefficients.

This logic holds when the $y$ -intercept, $\beta_o$, is fixed to zero, as is the case our study. Note that this logic also holds if error in $Y$ is not normally distributed, but remains unbiased (with mean =1).

The assumption of normally distributed $y$-direction percent errors is largely consistent with observed results in this study. However, Figure S3 demonstrates that there are two instances of stage 1 $y$-direction errors larger than +100%. Because there cannot be $y$-direction errors below -100%, this is a small deviation from the normality assumed in the above proof.

As a result, OLS remains an unbiased estimator if the sensor is indeed unbiased for all emission sizes, even with heteroskedastic noise. The edge effects introduced by non-detection events (which affect all linear regression specifications) are discussed further below, under **Bounding potential bias from fixing the $y$-intercept at zero**.

## X-direction errors are small compared to Y-direction errors

There are also errors in the metered emission rate due to metering error, flow variability, and uncertainty in gas composition. These errors are characterized in detail in [37], and constitute a maximum 95% confidence interval of ±13% of the metered value in this study, with a mean 95% confidence interval of ±11%. These uncertainties are nearly an order of magnitude smaller than errors in quantification accuracy, which have a standard deviation of 45%, shown in Table S5, and thus a 95% confidence interval of 88.2%.

Alternate regression specifications that account for error in $x$ values include standardized major axis and York regressions [60,61].

To evaluate the impact of $x$-direction errors on the linear fitted slope, we simulate 1,000 measurements between 0.2 t/h and 8 t/h with normally-distributed errors in the $x$- and $y$-directions. We simulate a data-generating process in which metering error (error in $x$) has a standard deviation of 5.5%, and thus a 95% confidence interval of 11%, and the simulated methane sensor is unbiased with errors (in $y$) with a standard deviation of 45%, with no correlation between errors in $x$ and errors in $y$.

We then fit OLS, York, and SMA regressions to this simulated dataset, with a nonzero intercept allowed. Regression results in Table S2 demonstrate that under these circumstances, OLS and York regressions produce estimates of the assumed true slope of 1 to within less than 0.5%. Notably, the fitted $y$ -intercept for OLS of - 0.027 [-0.317, 0.263] t/h is not statistically distinguishable from the true value of zero, but has larger uncertainty than the York regression. However, the York regression estimates the y-intercept at 0.011 [0.001, 0.021], statistically distinguishable from the true value of zero with 95% confidence, suggesting that a York regression can introduce a small amount of bias into the fitted intercept, albeit only of order 0.1% of the maximum allowable simulated value of 8 t/h.

Table S2. Linear fits to simulated controlled methane release testing with random error in both the instrument's quantification and the metering setup.

| Fit method | Slope [unitless] | Intercept [t/h] |
|---|---|---|
| OLS | 1.003 [0.934, 1.074] | - 0.027 [-0.317, 0.263] |
| York | 0.998 [0.993, 1.002] | 0.011 [0.001, 0.021] |
| SMA | 1.415 [1.354, 1.479] | -1.716 [-1.976, -1.468] |

SMA regression overestimates the true slope by over 40%, illustrating that this method will tend to introduce upward bias into the fitted slope, compensating with an unphysical and statistically significant negative intercept of -1.716 [-1.976, -1.468] t/h. The $R^2$ value of all three linear fits is 0.502.

We select OLS regression because it is widely used and understood and does not introduce bias into coefficient estimates. Although York regression is also essentially unbiased in fitted slope and produces tighter confidence intervals, it is less widely used and appears to introduce small artifacts into any fitted intercept.

These results demonstrate that our estimated level of uncertainty in the metered emission values does not introduce any noticeable bias into OLS.

## Rationale for fixing the intercept at zero

Using the relatively small dataset collected in this study, a standard OLS fit with an arbitrary y-intercept can produce linear fits with highly unphysical interpretation. If data exhibit an error profile that deviates from a symmetric error distribution around a line with an intercept fixed at zero, then a fitted line may have a substantial positive or negative intercept.

A negative intercept implies that below some emission size, a methane remote sensing system would see a methane sink rather than a source. A positive intercept implies that such a system would essentially always observe methane in every measurement regardless of the presence of actual emissions. In the satellite measurement dataset presented in this paper, standard floating-intercept OLS produces an intercept of 1.247 [0.105, 2.389] t/h and a correspondingly flattened slope of 0.577 [0.290, 0.865].

This non-physical intercept may in part simply be due to the relatively small sample size in this paper, which can introduce substantial variance into the fitted slope and intercept that deviate substantially from the underlying data-generating process.

To ensure a more physically-grounded interpretation of our linear fit results, we therefore fix the intercept at zero. The logic at the beginning of this section demonstrates that this will not introduce bias in a system with normally distributed percent errors in the y-direction.

However, our study only considers detected emissions when computing a line of best fit, following [18]. Thus, the false negatives (non-detected emissions) are not included in the linear fit. These results are interpretable as the quantification error profile for detected emissions. Including false negatives in the linear fit would introduce a different form of artifact into the

error distribution, with errors of -100% for all false negatives, instead of the normal error distribution in $y$ for a given $x$ value generally assumed in linear regression. Including false negatives in the regression could either increase or decrease the fitted OLS intercept, with false negatives for smaller emissions, like the two Sentinel-2 false negatives below 2 t/h, likely reducing the intercept and false negatives for larger emissions, such as the two Sentinel-2 false negatives at roughly 5 t/h, likely reducing the fitted slope and increasing the fitted intercept.

This means that either the omission or the inclusion of false negatives into the linear fit introduces an artifact into the error profile of detected emissions. If false negatives are excluded, as they are in this paper, then if a methane enhancement appears smaller than it actually is to a satellite-based methane sensing system, odds are increased that it will be designated as an artifact and not flagged as a detection. If the same methane enhancement appears larger than it actually is, odds are increased that it will be flagged as a detection. Thus, even the assumed normally distributed variation in methane quantification accuracy will introduce a tendency to under-detect emissions that, if quantified, would represent underestimates of the true emission rate.

## Bounding potential bias from fixing the y-intercept at zero

To bound the potential biasing effect of this aspect of the data-generating process, we use the above simulated methane emissions sensing dataset, but remove all emissions below 1-3 t/h before applying an OLS fit, simulating a system with a minimum detection threshold ranging from comparable in sensitivity to moderately less sensitive than Sentinel-2. Results of OLS fits with a floating intercept and with the intercept fixed at zero are shown in Table S3. For this simulation of 1,000 datapoints between 0.2 t/h and 8 t/h, treating all emissions estimates below 1 t/h as non-detections does not introduce statistically significant changes in the fitted slope and intercept, but does introduce a modest statistically significant increase the fitted slope by roughly 5%.

Table S3. OLS fit parameters to the above data with all simulated satellite estimates below Y t/h removed (treated as non-detections, emulating asymmetry in the quantification error profile introduced by the possibility of false negatives (i.e. missed detections). OLS floating intercept allows a nonzero y-intercept. OLS zero intercept does not. The N column lists the number of the original 1,000 datapoints included in the linear fit after excluding points below the min detection threshold listed in the first column.

| | OLS floating intercept | | OLS zero intercept | |
|---|---|---|---|---|
| Min detection threshold [t/h] | Slope [unitless] | Intercept [t/h] | Slope [unitless] | N |
| 1 | 1.026 [0.956, 1.097] | 0.15 [-0.198, 0.502] | 1.054 [1.025, 1.083] | 842 |
| 2 | 1.002 [0.913, 1.090] | 0.522 [0.053, 0.991] | 1.094 [1.063, 1.124] | 668 |
| 3 | 0.913 [0.797, 1.029] | 1.368 [0.706, 2.031] | 1.143 [1.110, 1.176] | 553 |

A larger minimum detection threshold of 2 t/h or 3 t/h results in a substantially larger intercept in the floating intercept cases, 0.522 [0.053, 0.991] t/h and 1.368 [0.706, 2.031] t/h, respectively, while the fitted slope remains statistically indistinguishable from the correct underlying value of

1. In these cases, fixing the y-intercept at zero introduces an upward bias in the slope of roughly 9% [6%, 12%] and 14% [11%, 18%], respectively.

The data in this study are probably closest to, but not identical to, the case with a minimum detection threshold of 1 t/h, suggesting that applying OLS with the y-intercept fixed at zero may bias the slope upward by roughly 5%.

# S5 Determining what satellites would see in the New Mexico Permian Basin

To estimate total methane emissions that a satellite with a given minimum detection limit, $\gamma$, would see in the field, we use a dataset of emissions detected during a comprehensive aerial survey of the New Mexico Permian Basin [4]. We compute total emissions from the survey with reported magnitude greater than or equal to $\gamma$ as a fraction of total estimated emissions in the surveyed area. Note that the survey covered only 91.2% of assets in the region and emissions estimates computed this way do not account for emissions from the remaining 8.8% of uncovered assets [4]. To account for this, we estimate total emissions in the surveyed area as the 194 t/h computed for the full New Mexico Permian times 91.2%, or 177 t/h. For simplicity, we assume all surveyed assets were covered four times, the average number across the survey when converting raw detections into persistence-averaged source-level emissions [4]. In practice, this equates to computing total detected emissions volume that a satellite of a given sensitivity would detect, treating all measurements as independent even if they were conducted at the same site, then dividing this total by four. Replication code is available in the main GitHub repository.

# S6 Supplementary results

## S6.1 Supplementary regression results

To estimate the overall quantification accuracy, goodness of fit, and error distribution of all quantified methane emission estimates, we apply a linear regression. For reasons described in the previous section, we fix the $y$-intercept at zero in the regression, shown in Eq. (11).

$$y = \beta x \tag{11}$$

Where $x$ is the mean metered emission rate, and y is the central emissions estimate provided by participating teams. These $x$ and $y$ values correspond to the markers in Figure 3.

The regression only includes quantified emissions, and does not include emissions that were not detected. We do this to assess the error distribution of detected emissions.

Table S4. Regression results for stages 1 and 2 based on the fixed-intercept ordinary least squares regression in Eq. (11).

| | Stage 1 | Stage 2 |
|---|---|---|
| $B$ | 0.855 [0.715, 0.889] | 1.0043 [0.889, 1.120] |
| Standard error | 0.069 | 0.057 |
| t-statistic | 12.474 | 17.761 |

| | | |
|---|---|---|
| No. Observations | 32 | 32 |
| Degrees of freedom (Residuals) | 31 | 31 |
| Degrees of freedom (Model) | 1 | 1 |
| Uncentered $R^2$ | 0.834 | 0.911 |
| Adjusted $R^2$ | 0.829 | 0.908 |
| F-statistic | 155.6 | 315.4 |

$R^2$ values are presented in uncentered format due to the absence of a y-intercept term in the regression specification. As a result, these $R^2$ values are not directly comparable with the centered $R^2$ values produced in regressions with a y-intercept.

Note that these regressions treat each estimate from each team and satellite as independent and identically distributed observations. This aggregation is necessary to produce a meaningful regression due to the small sample size for each satellite and team, but the results of this analysis should be treated as a rough illustration of the general capabilities of the participating satellites and teams as a whole. Detailed characterization of the quantification accuracy from individual satellites and teams will require more datapoints.

## S6.2 Error statistics by satellite and team

Table S5. Summary statistics of quantified (non-zero) emissions by satellite, across all teams.

| | | Stage 1 | | | | Stage 2 | | | |
|---|---|---|---|---|---|---|---|---|---|
| | Count | Min | Mean | Max | $\sigma$ | Min | Mean | Max | $\sigma$ |
| GHGSat-C2 | 3 | -17% | -4% | 13% | 16% | -8% | 8% | 28% | 18% |
| Landsat 8 | 5 | -57% | -29% | 7% | 25% | -34% | -11% | 20% | 21% |
| PRISMA | 6 | -20% | 27% | 110% | 49% | 7% | 44% | 64% | 20% |
| Sentinel-2 | 16 | -48% | 7% | 103% | 48% | -35% | 19% | 131% | 43% |
| WV3 | 2 | -68% | -32% | 3% | 50% | -60% | -19% | 21% | 58% |

Table S6. Summary statistics of quantified (non-zero) emissions by team, across all satellites.

| | | Stage 1 | | | | Stage 2 | | | |
|---|---|---|---|---|---|---|---|---|---|
| | Count | Min | Mean | Max | $\sigma$ | Min | Mean | Max | $\sigma$ |
| GHGSat | 3 | -17% | -4% | 13% | 16% | -8% | 8% | 28% | 18% |
| Harvard | 5 | -45% | -4% | 48% | 41% | -21% | 18% | 58% | 31% |
| Kayrros | 10 | -68% | 8% | 103% | 52% | -60% | 24% | 131% | 52% |
| SRON | 4 | -57% | -43% | -31% | 12% | -35% | -28% | -10% | 12% |
| LARS | 10 | -28% | 17% | 110% | 48% | -16% | 26% | 65% | 31% |

## S6.3 Aggregate error statistics and effect of metered time averaging period

Table S7. Summary statistics of the percent error of estimated emission rates, as well as stage 1 wind speed error. Compares central estimates with 5-minute mean measured emissions. Note that although the standard deviation of the percent error distribution falls slightly after wind unblinding in stage 2, the inter-quartile range between the 25th and 75th percentiles of the error distribution is larger in stage 2. Thus, although by some metrics the linear fit improves using 10-m wind measurements, doing so in this case leads to larger percent errors for some smaller emission rates, leading to a similar overall percent error distribution. Note that the stage 1 percent error distribution does not change appreciably if the 300-second time average is replaced with 60 seconds or 600 seconds.

| **Metric** | **Stage 1 (fully blind, 300s meter avg)** | **Stage 2 (measured wind)** | **Wind speed** | **Stage 1 (60s meter avg)** | **Stage 1 (600s meter avg)** |
|---|---|---|---|---|---|
| **Mean** | 2% | 16% | -19% | 2% | 2% |
| **Standard deviation** | 45% | 39% | 25% | 46% | 45% |
| **Min** | -68% | -60% | -52% | -67% | -68% |
| **P25** | -28% | -12% | -39% | -28% | -29% |
| **P50 (median)** | -7% | 13% | -22% | -8% | -6% |
| **P75** | 16% | 45% | -3% | 17% | 17% |
| **Max** | 110% | 130% | 60% | 113% | 108% |
| **Inter-quartile range (P75-P25)** | 45% | 57% | 35% | 45% | 46% |

## S6.4 Detection summary

Table S8. Detection results by satellite and team. A tabular representation of Figure 1.

| Satellite | Team | # True positive | # False negative | # True negative | # false positive | # Filtered retrieval | # Not tasked | Total |
|---|---|---|---|---|---|---|---|---|
| GHGSat-C2 | GHGSat | 3 | 0 | 0 | 0 | 0 | 2 | 5 |
| Landsat 8 | LARS | 2 | 0 | 0 | 0 | 0 | 0 | 2 |
| Landsat 8 | Kayrros | 2 | 0 | 0 | 0 | 0 | 0 | 2 |
| Landsat 8 | SRON | 1 | 0 | 0 | 0 | 1 | 0 | 2 |
| PRISMA | LARS | 3 | 0 | 0 | 0 | 0 | 1 | 4 |
| PRISMA | Kayrros | 3 | 0 | 0 | 0 | 0 | 1 | 4 |
| Sentinel-2 | LARS | 4 | 1 | 1 | 0 | 1 | 0 | 7 |
| Sentinel-2 | Kayrros | 4 | 1 | 1 | 0 | 1 | 0 | 7 |
| Sentinel-2 | SRON | 3 | 2 | 0 | 0 | 2 | 0 | 7 |
| Sentinel-2 | Harvard | 5 | 0 | 1 | 0 | 1 | 0 | 7 |
| WV3 | LARS | 1 | 0 | 0 | 0 | 0 | 0 | 1 |
| WV3 | Kayrros | 1 | 0 | 0 | 0 | 0 | 0 | 1 |
| Total | - | 32 | 4 | 3 | 0 | 6 | 4 | 49 |

Table S9. Ground truth for detection by satellite. Includes the count of non-zero emissions as well as zero-emission controls given to each satellite for all measurements (all instances in which the satellite passed overhead). Only Sentinel-2 was given a zero.

| Satellite | # Non-zero | # Zero |
|---|---|---|
| GHGSat-C2 | 5 | 0 |
| Landsat 8 | 2 | 0 |
| PRISMA | 4 | 0 |
| Sentinel-2 | 6 | 1 |
| WV3 | 1 | 0 |

## S6.5 Supplementary figures

Underlying data and code to reproduce these figures are available in the data and code repository for this paper, particularly in "matchedDF_Satellites_230130.csv".

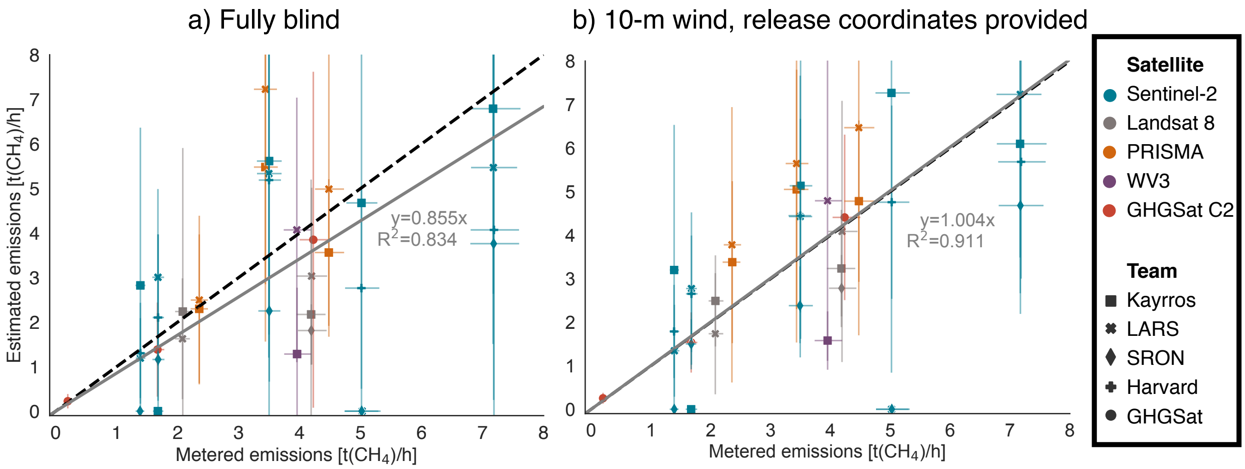


Figure S1. Stage 1 (fully blind) and stage 2 (with measured 10-m wind speed and direction as well as the precise coordinates of the release point) quantification estimates with 95% confidence intervals. The black dashed line denotes exact 1:1 agreement. Fitted slope and uncentered $R^2$ shown for an ordinary least squares fit with the intercept fixed at zero.

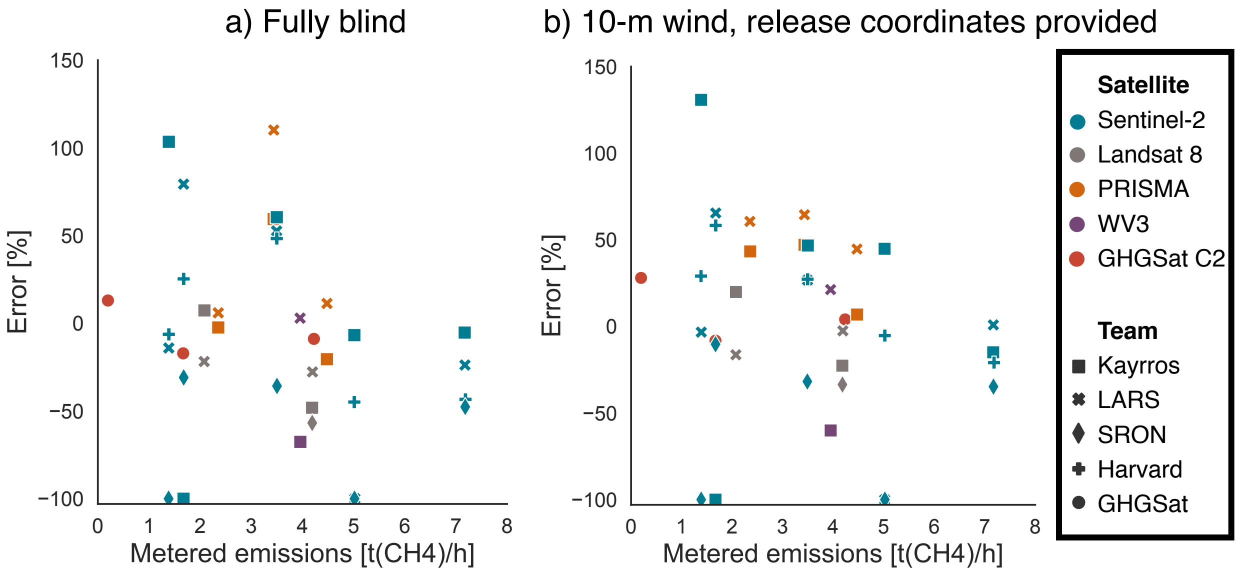


Figure S2. Percent error for stage 1 (fully blind) and stage 2 (with measured 10-m wind speed and direction as well as the precise coordinates of the release point).

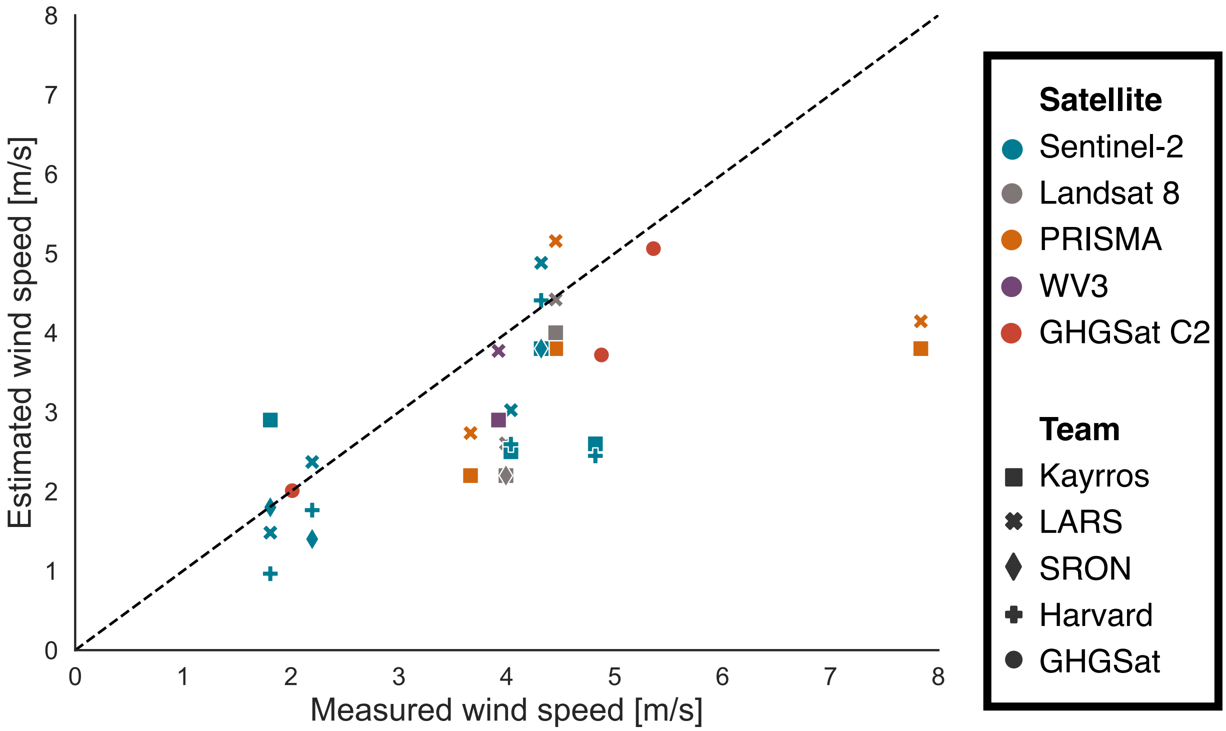


Figure S3. Parity chart of wind speed estimates used by teams in stage 1 compared with 1-minute averages from the 10-m ultrasonic anemometer. Only SRON provided low and high uncertainty bounds for wind speed estimates, not shown in this plot. Only includes wind speeds for nonzero quantified emissions, as some teams did not report wind speeds for non-detects. The black dashed line denotes exact 1:1 agreement.

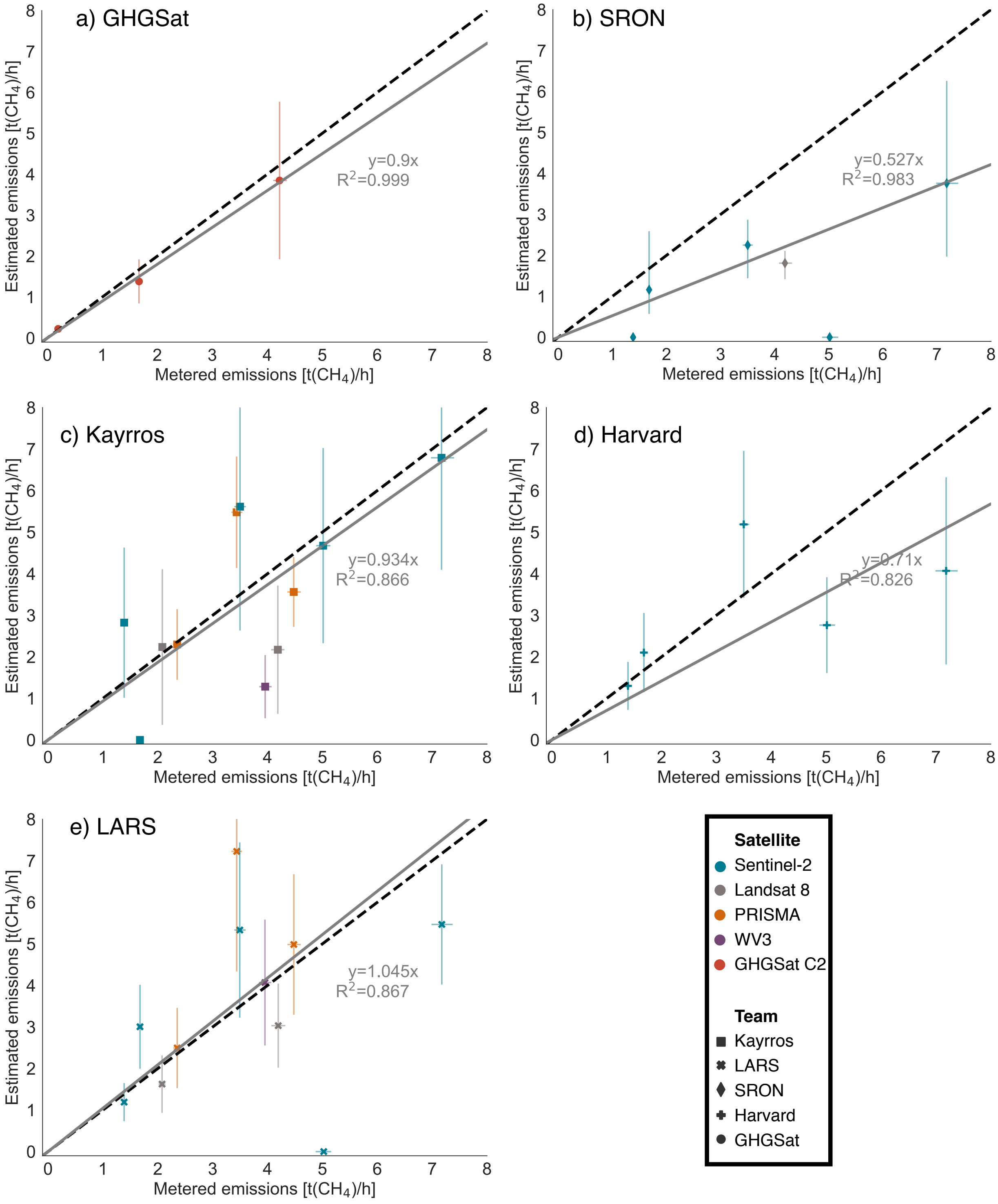


Figure S4. Stage 1 quantification performance by team across all satellites, with 1-sigma X and Y errorbars. The black dashed line denotes exact 1:1 agreement. Fitted slope and uncentered $R^2$ shown for an ordinary least squares fit with the intercept fixed at zero.

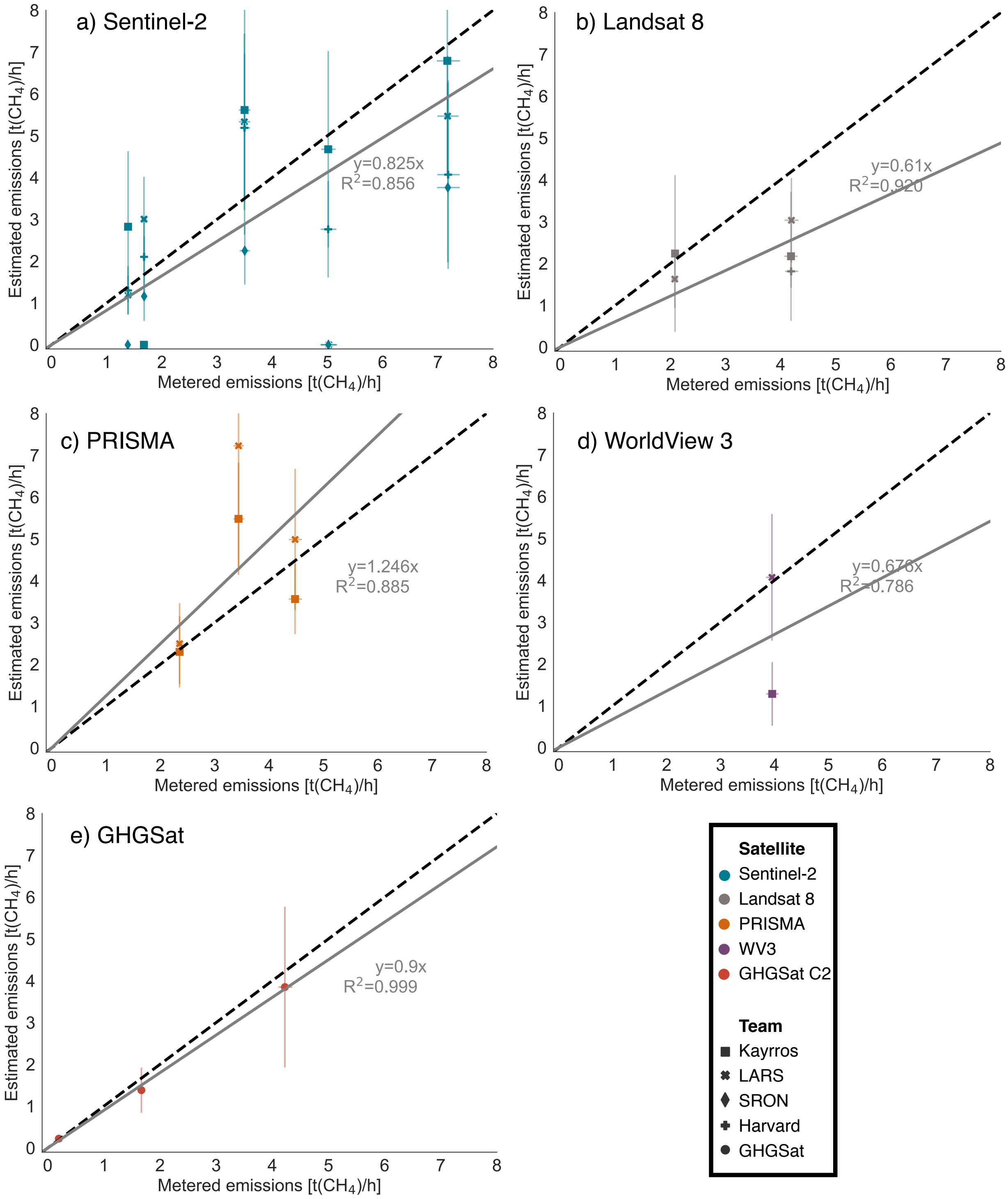


Figure S5. Stage 1 quantification performance by satellite across all teams, with 1-sigma X and Y errorbars. The black dashed line denotes exact 1:1 agreement. Fitted slope and uncentered $R^2$ shown for an ordinary least squares fit with the intercept fixed at zero.

The following are masked and unmasked methane retrieval images from each of the participating teams. Masking refers to the process of identifying a methane plume and differentiating its outline from its surroundings. Submitting these images was optional, and not all teams submitted all images for retrievals they conducted. Note the level of variability in unmasked scenes across teams operating with precisely the same spectral data.

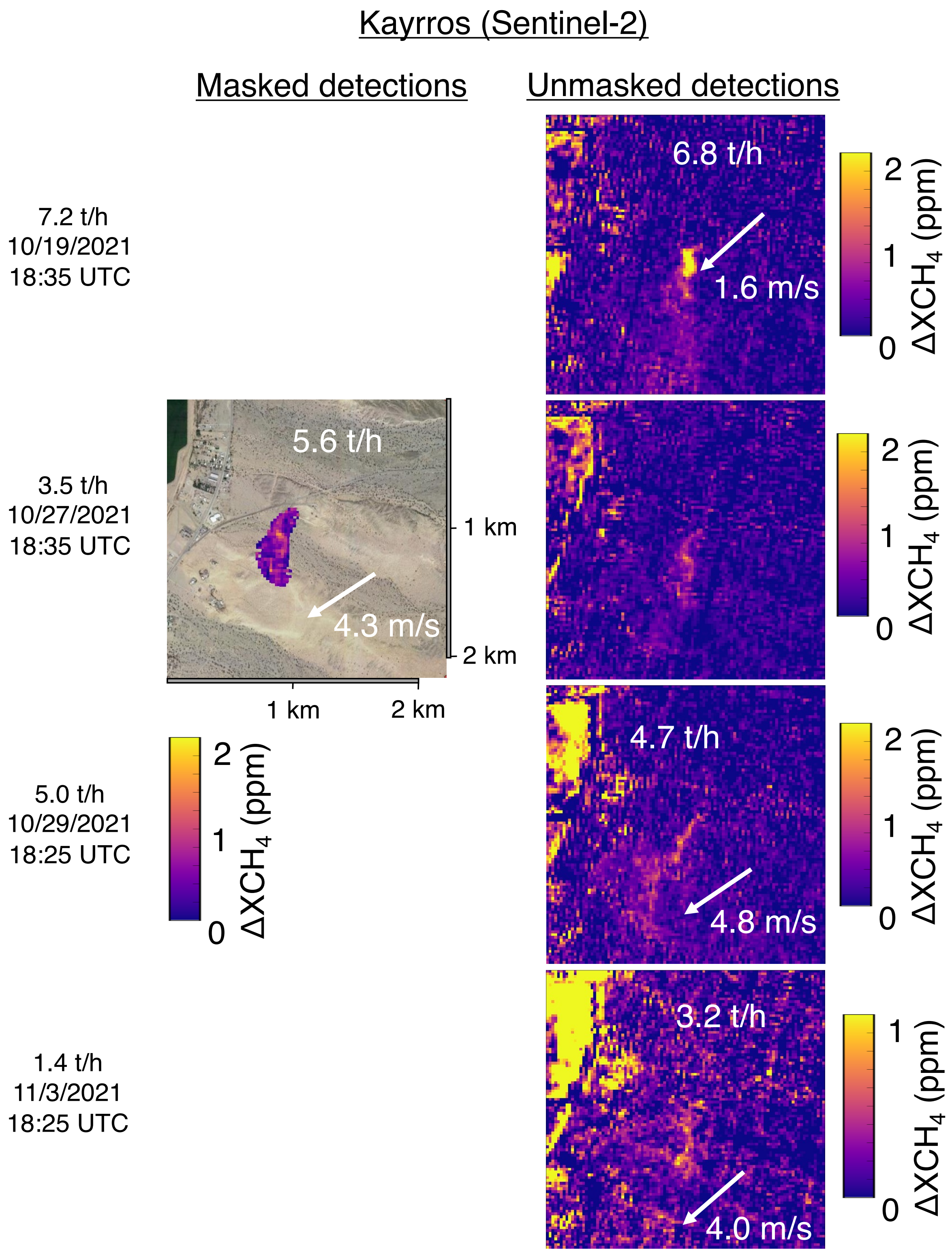


Figure S6. Provided masked and unmasked methane enhancement estimates from Kayrros for Sentinel-2 retrievals. Note that only October 27 was provided in masked form. Metered 5-minute average emission rate shown in black text. Estimated emission rate and measured 1-minute average wind speed and direction inset in white text. Surface imagery © 2021 Google Earth.

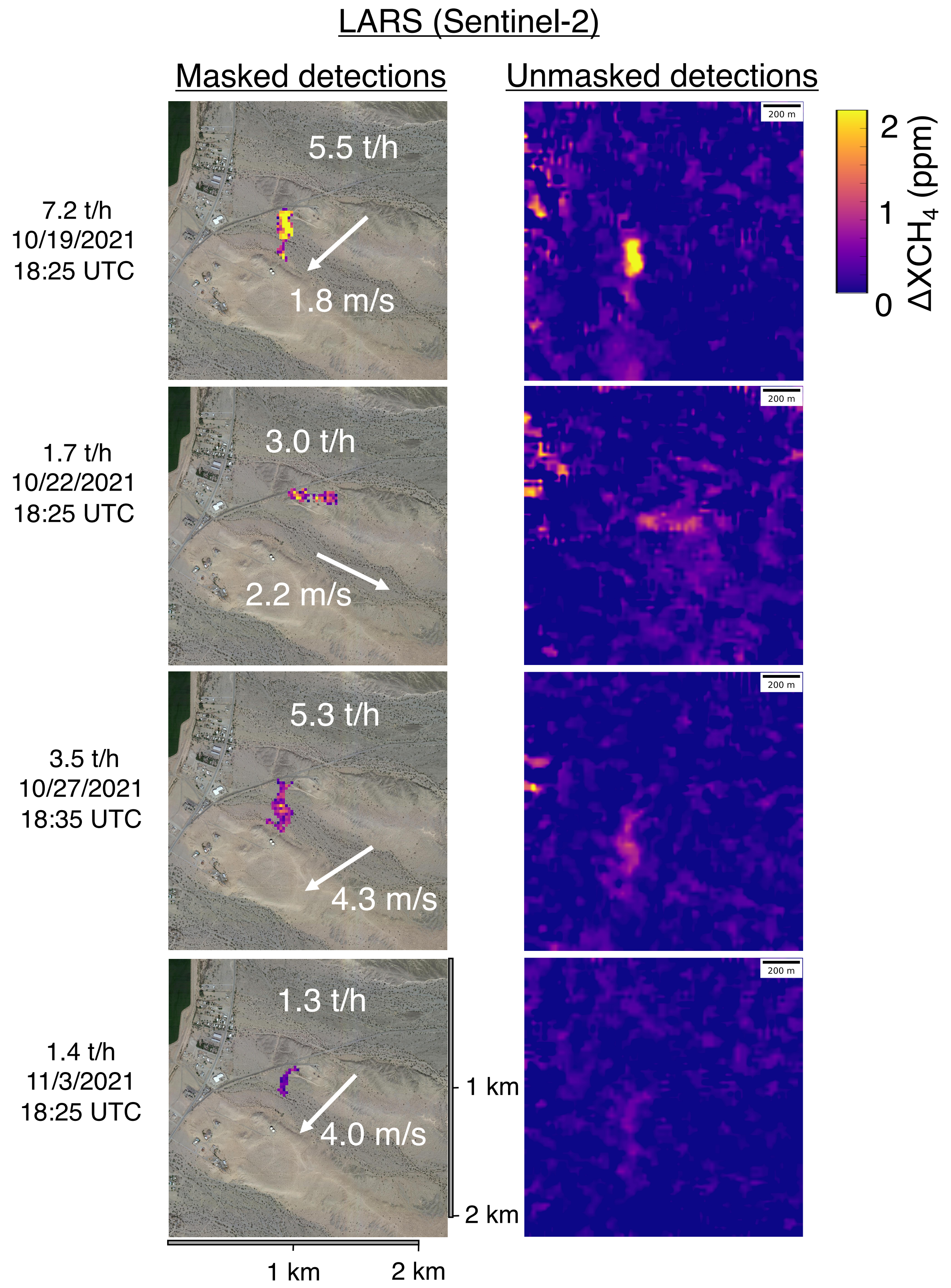


Figure S7. Provided masked and unmasked methane enhancement estimates from LARS for Sentinel-2 retrievals. Unmasked images presented are after filtering, immediately before masking. Metered 5-minute average emission rate shown in black text. Estimated emission rate and measured 1-minute average wind speed and direction inset in white text. Surface imagery © 2021 Google Earth.

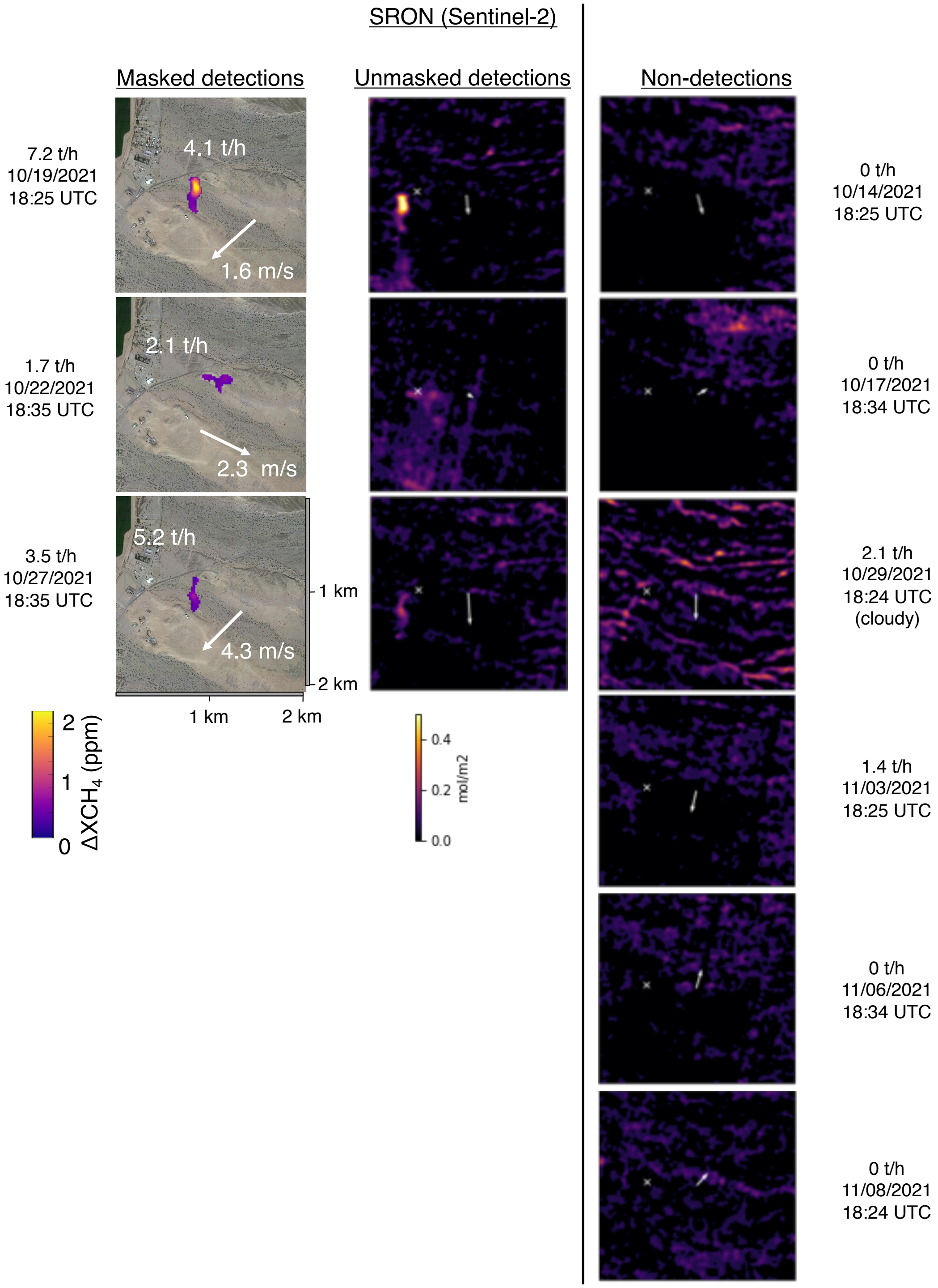


Figure S8. Provided masked and unmasked methane enhancement estimates from SRON for the Sentinel-2 retrievals, including several days in which no testing was conducted (October 14, November 6, and November 8, 2021), as well as non-detection instances and filtered retrievals, including a cloudy background on October 29. Note the use of differing scales in the masked and unmasked images. Metered 5-minute average emission rate shown in black text. Estimated emission rate and measured 1-minute average wind speed and direction inset in white text. Surface imagery © 2021 Esri, NASA, NGA, USGS, FEMA | County of Riverside, California State Parks, Esri,

HERE, Garmin, SafeGraph, GeoTechnologies, Inc., METI/NASA, USGS, Bureau of Land Management, EPA, NPS, US Census Bureau, USDA | Maxar, Google Earth.

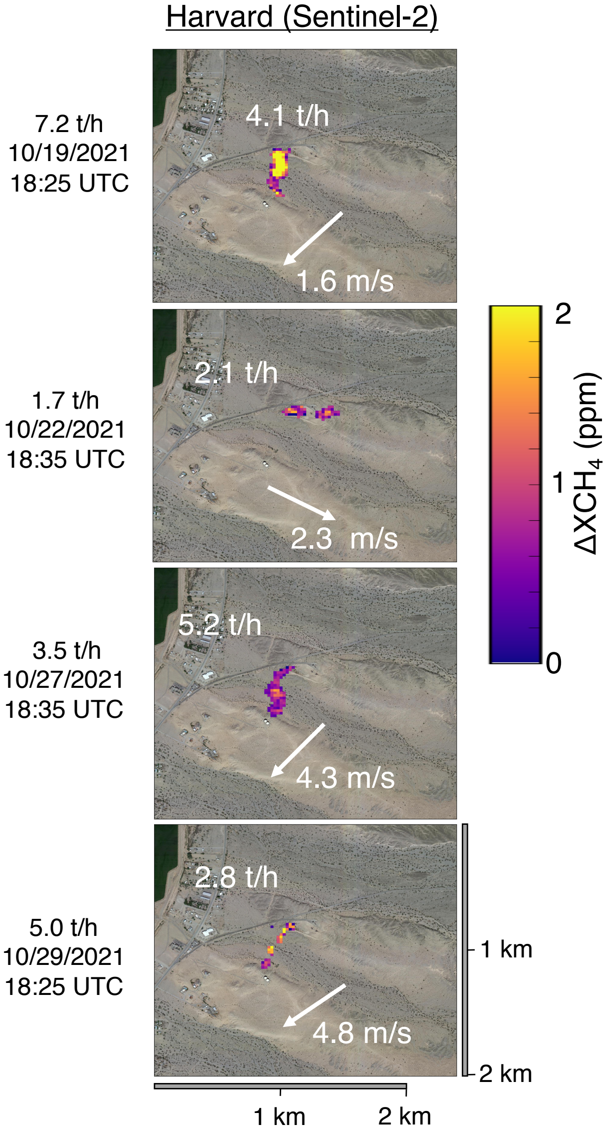


Figure S9. Provided masked methane enhancement estimates from Harvard for the Sentinel-2 retrievals. Metered 5-minute average emission rate shown in black text. Estimated emission rate and measured 1-minute average wind speed and direction inset in white text. Surface imagery © 2021 Google Earth.

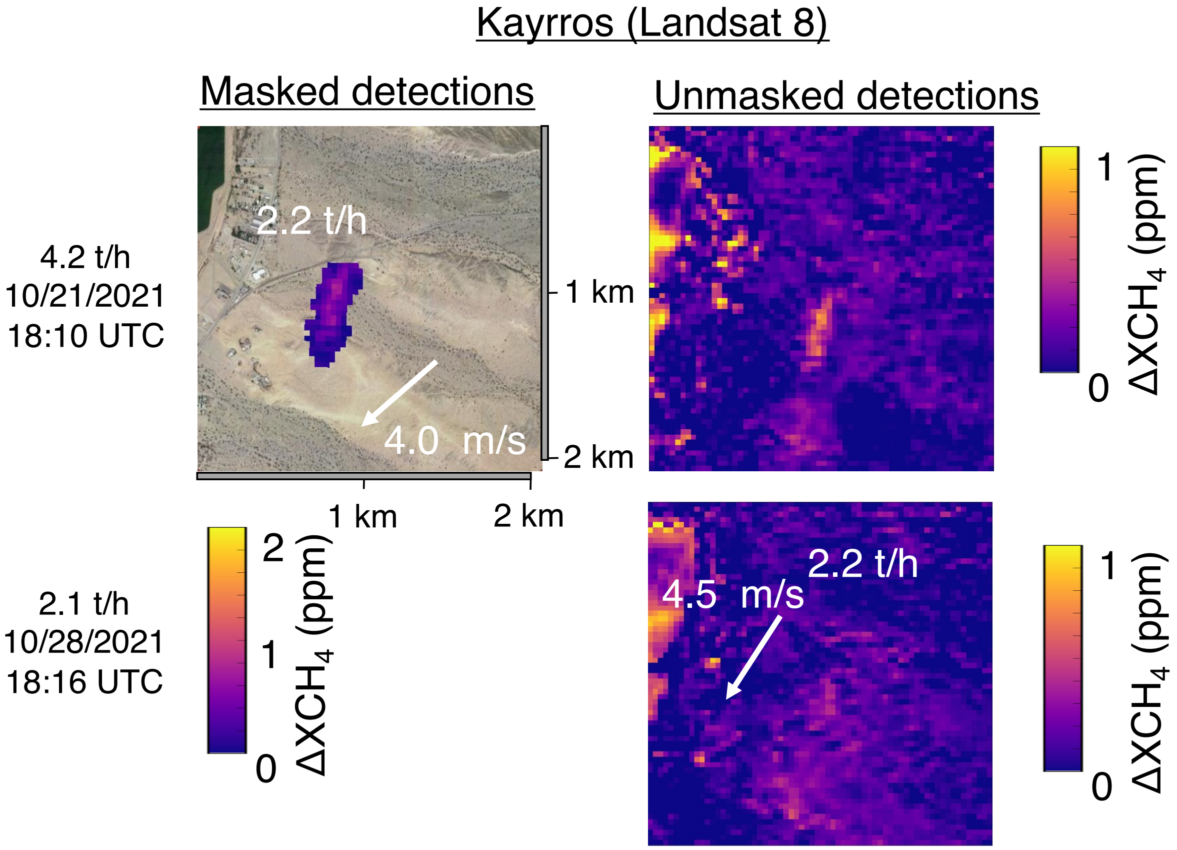


Figure S10. Provided masked and unmasked methane enhancement estimates from Kayrros for Landsat 8 retrievals. Note that only October 21 was provided in masked form. Metered 5-minute average emission rate shown in black text. Estimated emission rate and measured 1-minute average wind speed and direction inset in white text. Surface imagery © 2021 Google Earth.

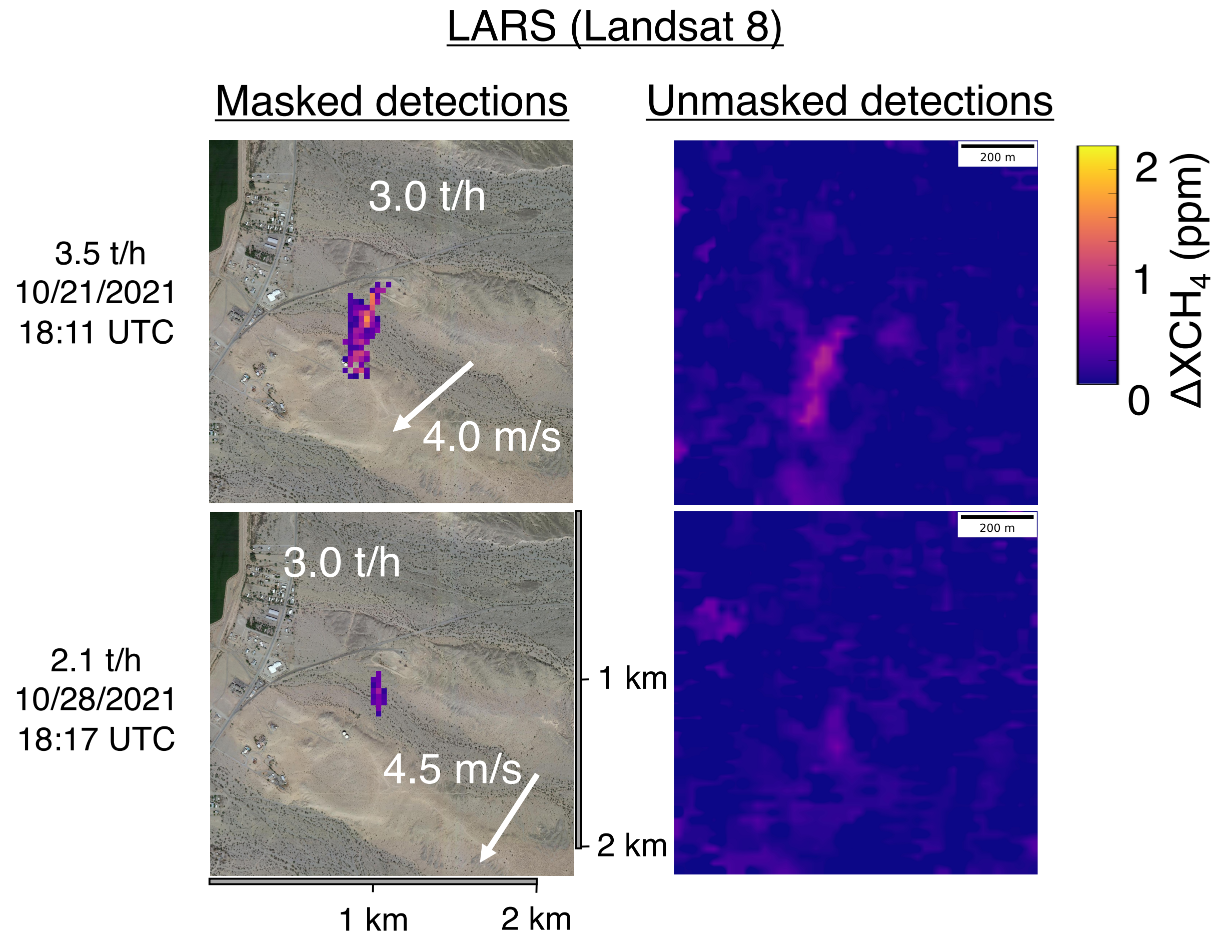


Figure S11. Provided masked and unmasked methane enhancement estimates from LARS for the Landsat 8 retrievals. Unmasked images presented are after filtering, immediately before masking. Metered 5-minute average emission rate shown in black text. Estimated emission rate and measured 1-minute average wind speed and direction inset in white text. Surface imagery © 2021 Google Earth.

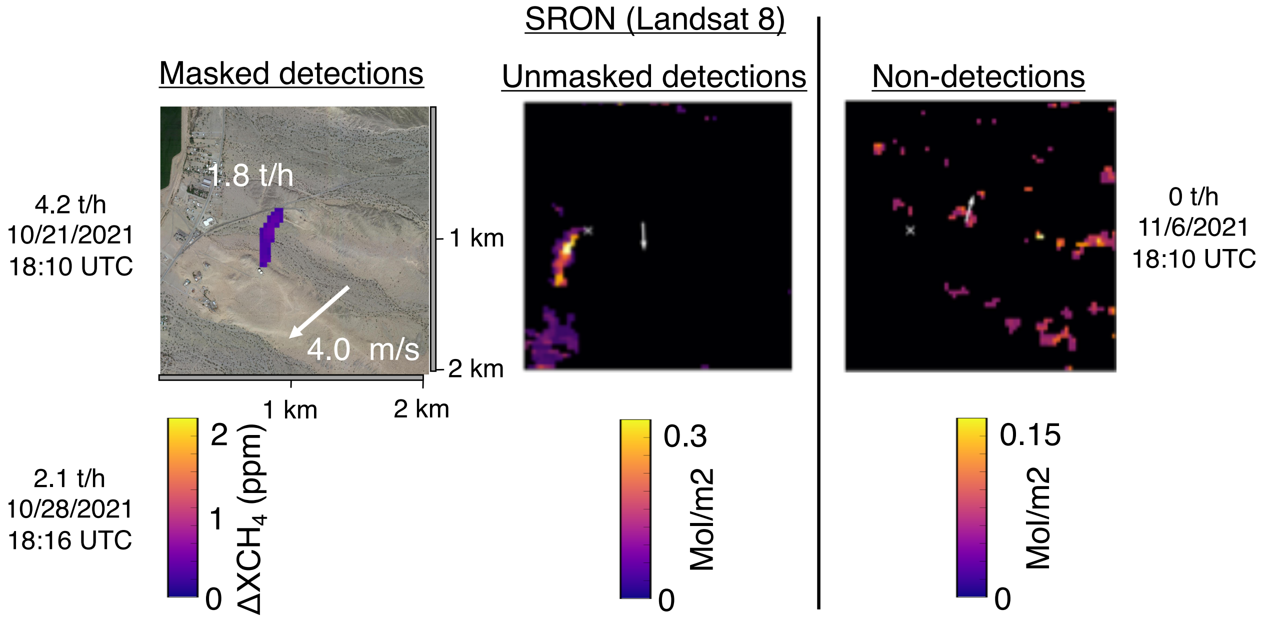


Figure S12. Provided masked and unmasked methane enhancement estimates from SRON for the Landsat 8 retrievals, including one day on which no testing was conducted (November 6, 2021). Note the use of differing scales in the masked and unmasked images. Metered 5-minute average emission rate shown in black text. Estimated emission rate and measured 1-minute average wind speed and direction inset in white text. Surface imagery © 2021 Esri, NASA, NGA, USGS, FEMA | County of Riverside, California State Parks, Esri, HERE, Garmin, SafeGraph, GeoTechnologies, Inc., METI/NASA, USGS, Bureau of Land Management, EPA, NPS, US Census Bureau, USDA | Maxar, Google Earth.

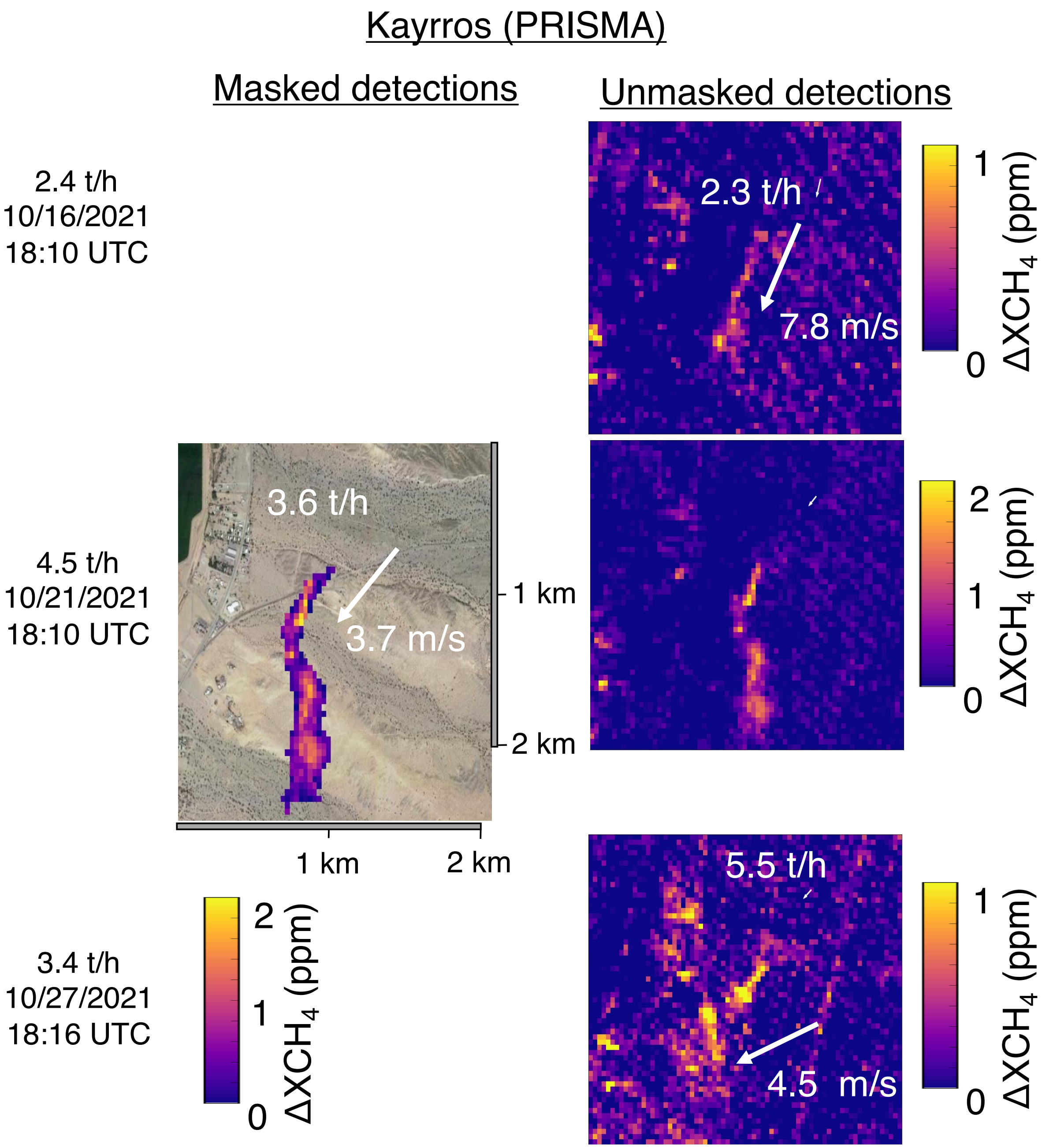


Figure S13. Provided masked and unmasked methane enhancement estimates from Kayrros for PRISMA retrievals. Note that only October 21 was provided in masked form. Metered 5-minute average emission rate shown in black text. Estimated emission rate and measured 1-minute average wind speed and direction inset in white text. Surface imagery © 2021 Google Earth.

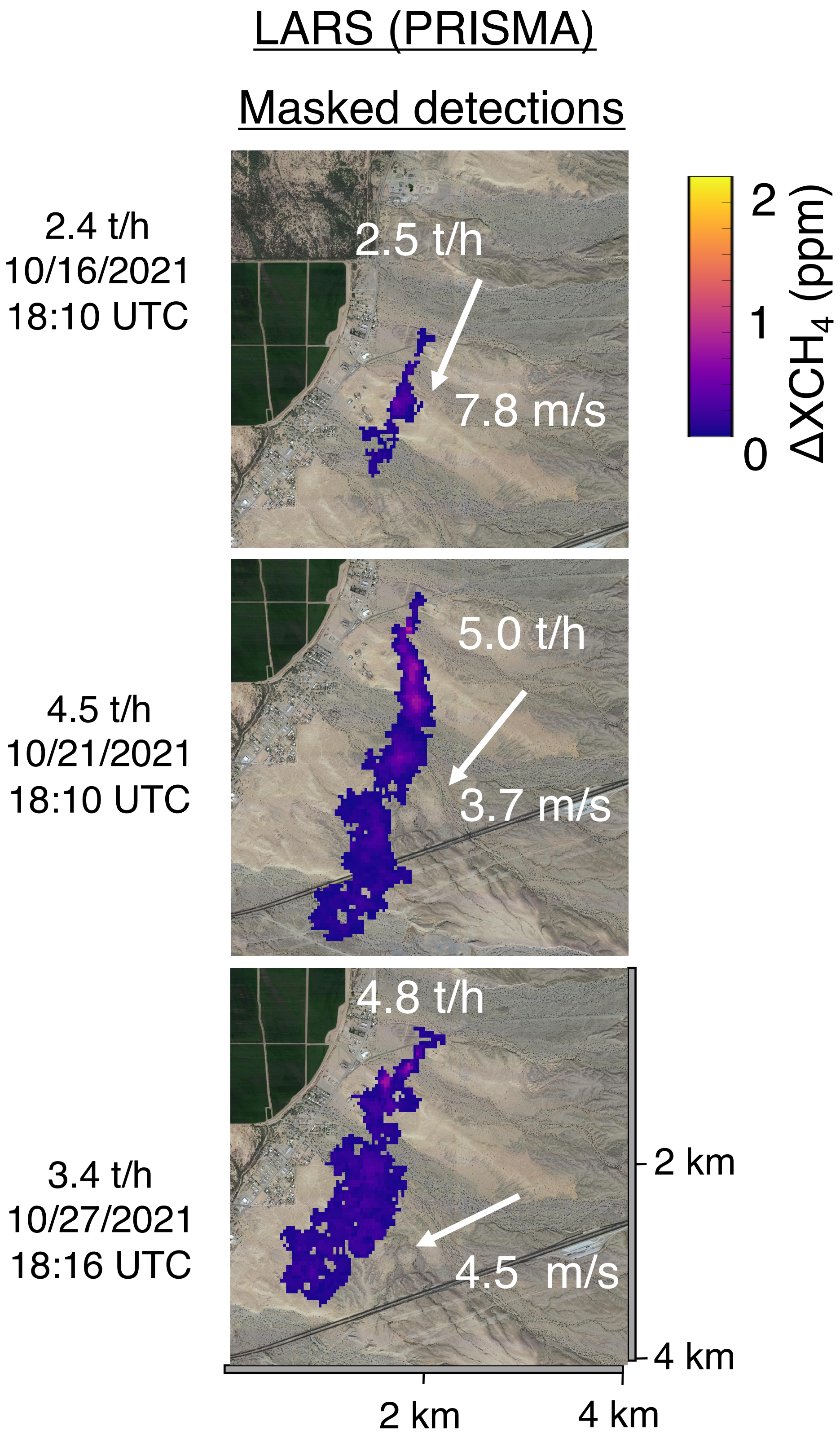


Figure S14. Provided masked methane enhancement estimates from LARS for the PRISMA retrievals. Providing unmasked images was optional in this test. Metered 5-minute average emission rate shown in black text. Estimated emission rate and measured 1-minute average wind speed and direction inset in white text. Surface imagery © 2021 Google Earth.

LARS provided only the masked image in Figure 2 for WorldView 3. As a result, we do not display a corresponding supplementary figure here.

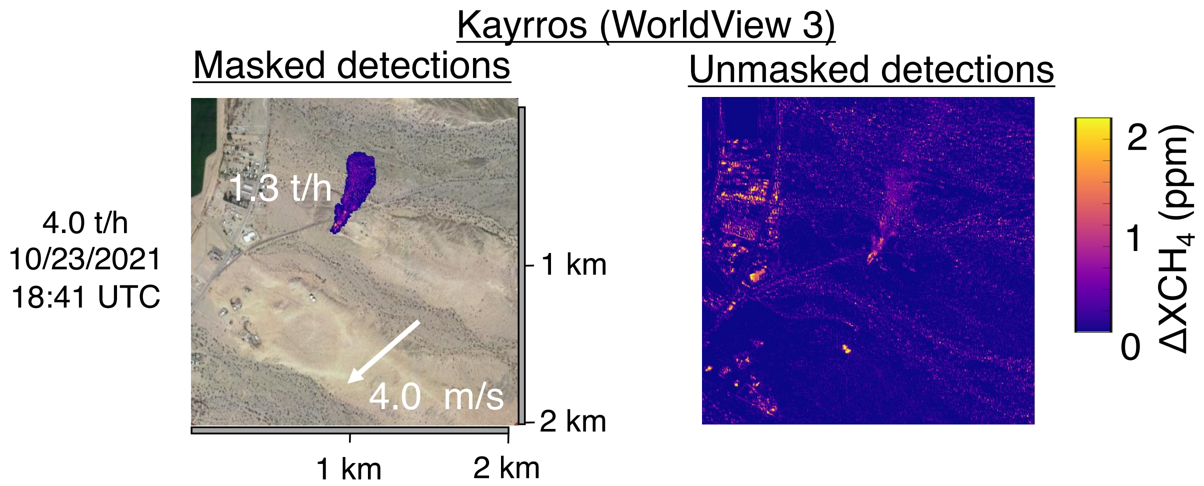


Figure S15. Provided masked and unmasked methane enhancement estimates from Kayrros for the WorldView 3 retrieval. Metered 5-minute average emission rate shown in black text. Estimated emission rate and measured 1-minute average wind speed and direction inset in white text. Surface imagery © 2021 Google Earth.

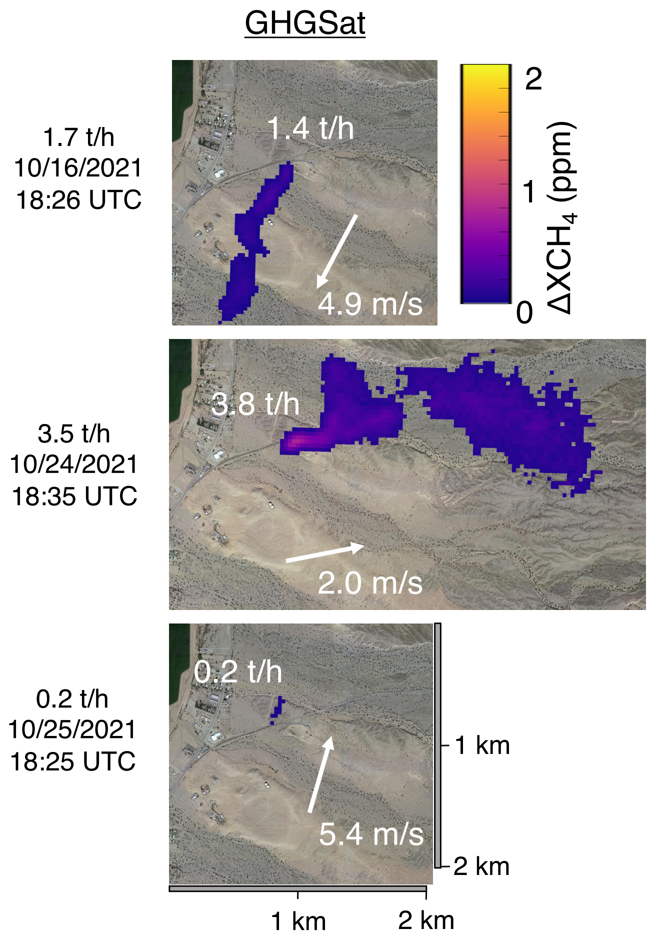


Figure S16. Provided masked methane enhancement estimates from GHGSat retrievals using the GHGSat-C2 satellite. Metered 5-minute average emission rate shown in black text. Estimated emission rate and measured 1-minute average wind speed and direction inset in white text. Surface imagery © 2021 Google Earth.

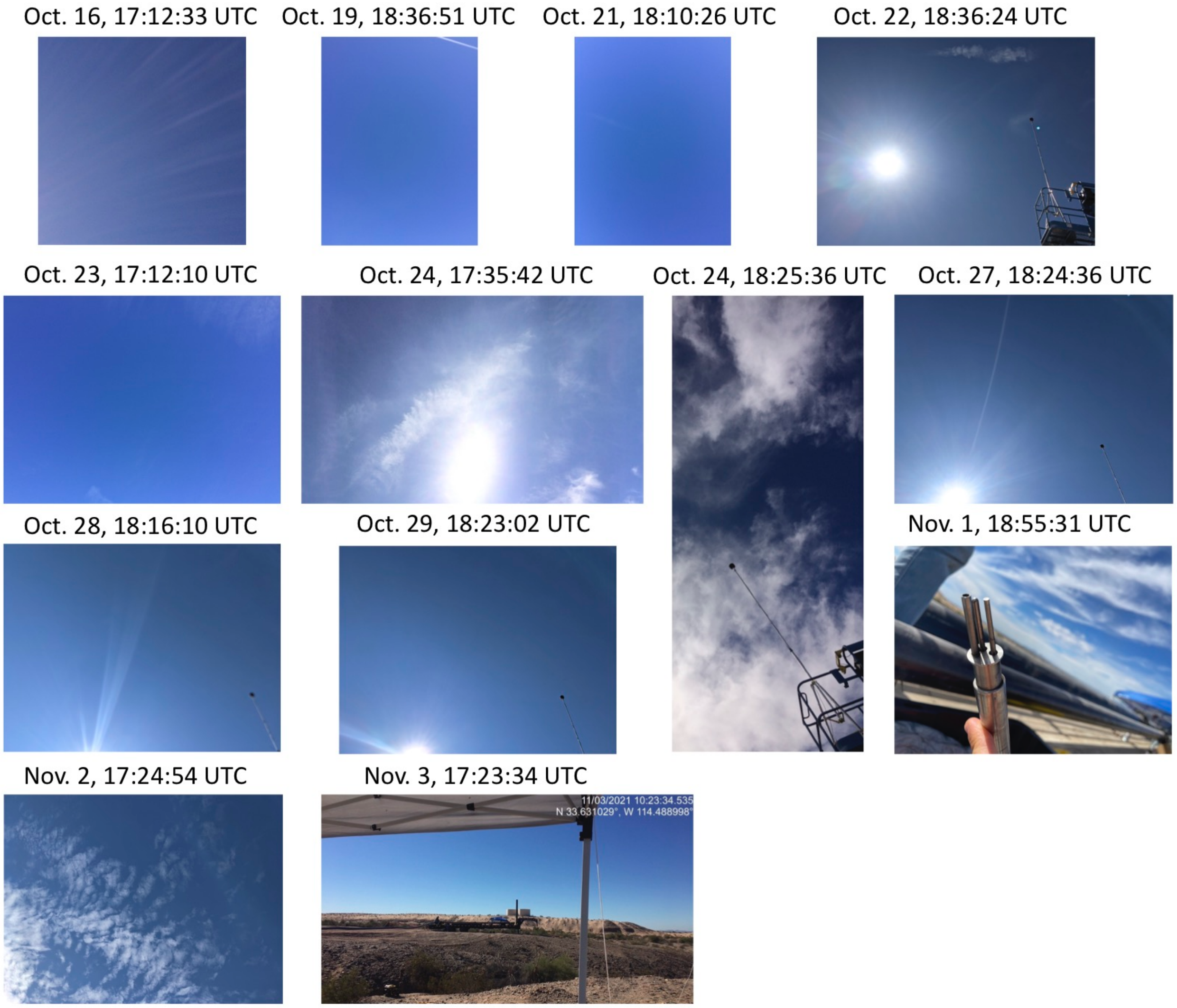


Figure S17. Photographs of the sky above the release site, taken by Stanford researchers near satellite overpass times. November 1 and November 3 photographs are the closest in time taken to the time of satellite overpass on days in which researchers did not take intentional sky photographs. The October 22, 24, 27, 28, and 29 photographs all include the nearby ultrasonic anemometer.
